# Cyclometalated Rhodium(III) Polypyridyl Complexes with Anti-Cancer Stem Cell Activity

**DOI:** 10.1021/acs.organomet.6c00018

**Published:** 2026-03-12

**Authors:** Hao Ren, Kuldip Singh, Kogularamanan Suntharalingam

**Affiliations:** School of Chemistry, 4488 University of Leicester, Leicester LE1 7RH, U.K.

## Abstract

Investigations into the anticancer stem cell (CSC) properties of rhodium complexes are extremely rare. Here we report the synthesis, characterization, photophysical properties, and *in vitro* anti-CSC activity of a series of cyclometalated rhodium­(III)-polypyridyl complexes **1**-**4**. The 4,7-diphenyl-1,10-phenanthroline-bearing complex **4** exhibits activity against monolayer- and three-dimensionally cultured breast CSCs and osteosarcoma stem cells in the sub-micromolar to low micromolar range, outperforming the metallodrug cisplatin and the established anti-CSC agent salinomycin. Our results suggest that the reported rhodium­(III) scaffold, containing cyclometalated 2,2′-(phenylmethylene)­dipyridine, could be a useful synthon for the future development of anti-CSC rhodium complexes.

## Graphical abstract

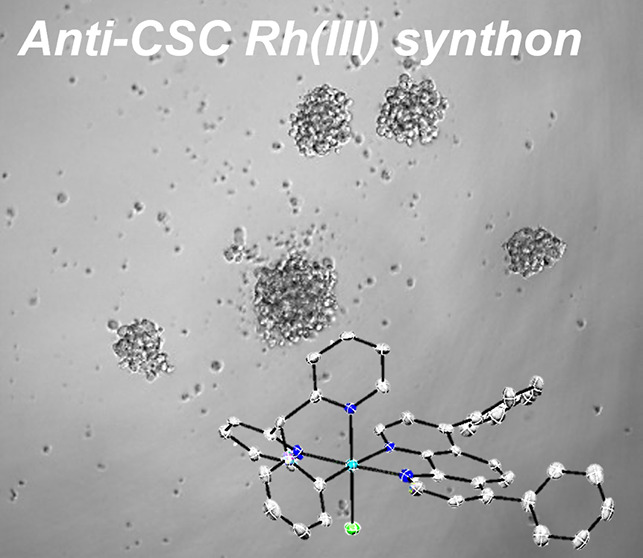

Metal complexes are an important component of modern-day chemotherapy.^1^ Four metal complexes (cisplatin, carboplatin, oxaliplatin, and arsenic trioxide) have been universally approved as chemotherapies for specific forms of cancer.^1b,2^ Despite their widespread use in the clinic, the current pool of anticancer metallodrugs have distinct disadvantages including side effects resulting from systematic toxicity and the inability to prevent relapse and metastasis.^1,3^ Systematic toxicity is a manifestation of poor stability, fast ligand exchange kinetics, and speciation in biological fluids.^3a^ Meanwhile, the inability of the metallodrugs to kill cancer stem cells (CSCs) at their clinically administered doses is a contributing factor in their ineffectiveness in stopping relapse and metastasis.^4^ CSCs are a subpopulation of tumor cells with the capacity to differentiate and self-renew and are inherently less susceptible to chemotherapies as they tend to have slow cell cycle profiles and most chemotherapies selectively kill proliferating cells.^5^ Over the past decade, we and others have explored the anti-CSC properties of several classes of metal complexes in *in vitro* and *in vivo* systems.^4,6^ There is a large body of work on the anti-bulk cancer cell activity of rhodium­(I/II/III) complexes,^7^ but only one rhodium complex has been studied in CSC models.^8^ In this study we sought to expand this knowledge space by investigating the anti-CSC properties of a series of cyclometalated rhodium­(III)-polypyridyl complexes (**1**-**4**, Figure 1a). The low-spin d^6^ electron configuration associated with rhodium­(III) complexes was envisaged to confer stability in biologically relevant solutions, whereas the N∧N∧C-cyclometalated and polypyridyl ligands were predicted to endow sufficient lipophilicity to facilitate CSC uptake.

**Figure 1:**
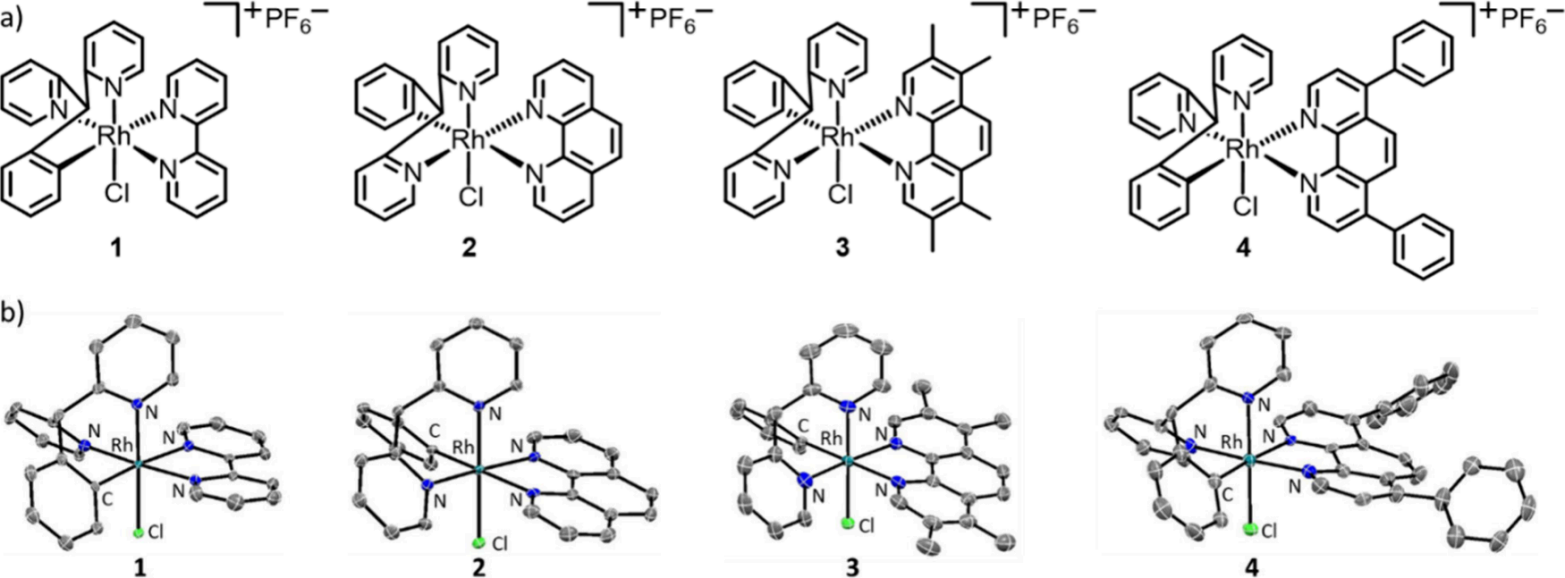
(a) Chemical structures of the cyclometalated rhodium­(III)-polypyridyl complexes **1**-**4** under investigation in this study. (b) X-ray structures of **1**-**4** containing a distorted octahedral rhodium­(III) center bound to 2,2′-(phenylmethylene)­dipyridine, the corresponding polypyridyl ligand, and a chloride ligand. The ellipsoids are set at the 50% probability level. Hydrogen atoms, the hexafluorophosphate counter-anion, and any cocrystallizing solvents have been omitted for clarity.

The cyclometalated rhodium­(III)-polypyridyl complexes **1**-**4** investigated in this study are depicted in Figure 1a. To synthesize **1**-**4**, 2,2′-(phenylmethylene)­dipyridine (**L** ^**1**^)^9^ was combined with RhCl_3_·3H_2_O in 2-methoxyethanol:water (3:1) and refluxed for 2 days, after which the appropriate polypyridyl ligand was added (2,2′-bipyridine for **1**, 1,10-phenanthroline for **2**, 3,4,7,8-tetramethyl-1,10-phenanthroline for **3**, and 4,7-diphenyl-1,10-phenanthroline for **4**) and the mixture was refluxed for a further 24 h. The solvent was then removed, and the resultant solid was converted to the corresponding hexafluorophosphate salt. Upon various solvent washing or recrystallization steps, pure **1**-**4** were isolated in diverse yields (13–80%) and characterized by ^1^H, ^13^C­{^1^H}, ^19^F­{^1^H}, and ^31^P­{^1^H} NMR spectroscopy, infrared spectroscopy, high-resolution ESI mass spectrometry, and elemental analysis (see Figures S1–S24). Single crystals of **1**-**4** suitable for X-ray diffraction studies were obtained by slow diffusion of diethyl ether into an acetonitrile or dichloromethane solution of **1**-**4** (CCDC 2518219–2518222, Figure 1b, and Tables S1–S2). Selected bond distances and bond angles for **1**-**4** are presented in Tables S3–S6. All of the rhodium­(III) complexes adopt distorted octahedral structures, where the central rhodium­(III) atom is bound to two nitrogen atoms and one carbon atom in 2,2′-(phenylmethylene)­dipyridine, two nitrogen atoms in the corresponding polypyridyl ligand, and a chloride atom. The Rh–N, Rh–C, and Rh–Cl bond distances determined for **1**-**4** are consistent with bond parameters for structurally similar rhodium­(III) complexes.^10^ Notably, in all complexes, the Rh–N_polypyridyl_ bond *trans* to carbon is longer than that *trans* to nitrogen. This phenomenon can be attributed to the greater *trans* effect of carbon compared to nitrogen (carbon is a stronger σ-donor than nitrogen).^11^

The photophysical properties of **1**-**4** were investigated in acetonitrile and water and are summarized in Tables S7–S8 (see Figures S25–S28 for the absorption and emission spectra). The rhodium­(III) complexes **1**-**4** displayed intense absorption bands between 250 and 325 nm, which are tentatively assigned to intra- and interligand charge transfer (ILCT) transitions involving 2,2′-(phenylmethylene)­dipyridine and the corresponding polypyridine ligands (Figures 2, S25, and S27).^9a,11,12^ Weaker shoulder bands between 325 and 400 nm are assigned largely to spin allowed and forbidden metal-to-ligand charge transfer (MLCT) or ligand-to-ligand charge transfer (LLCT) transitions.^9a,11,12^ Generally **1**-**4** exhibit weak emission in acetonitrile (λ_ex_ = 340 nm, Figure S26). In water, **1** and **2** are largely nonemissive, whereas **3** and **4** display broadened, structureless emission profiles with peak maxima around 390 and 460 nm (λ_ex_ = 340 nm, Figures 2 and S28). The quantum yields (Φ, %) of the rhodium­(III) complexes in acetonitrile and water **1**-**4** varied between 0.08 to 0.93 and were not directly related to the polypyridine ligand present (Tables S7–S8 and Figures S29–S30). The quantum yield values obtained for **1**-**4** are significantly lower than those reported for structurally related complexes containing iridium­(III).^9a,12^

**Figure 2:**
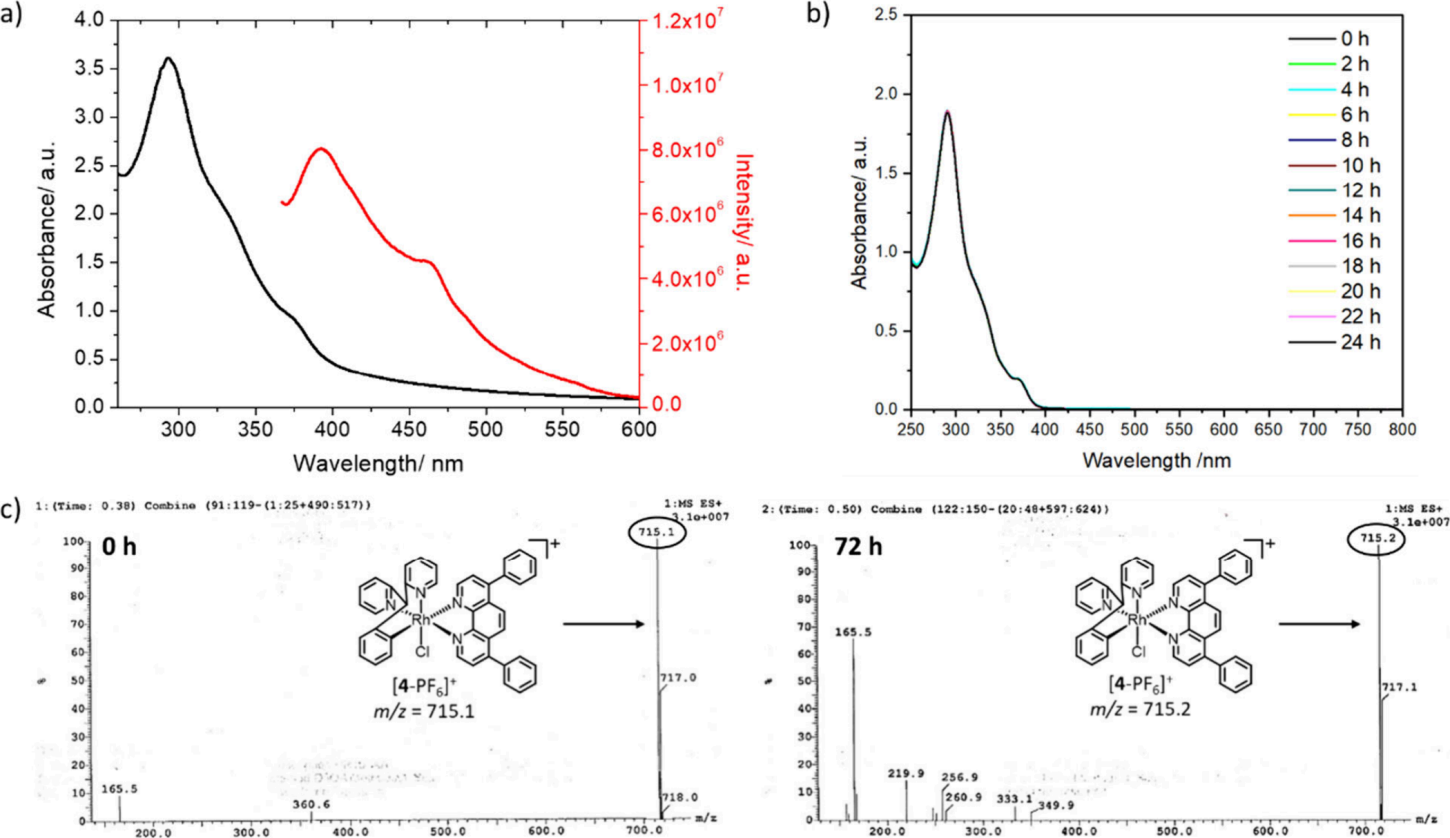
(a) UV–vis spectrum (black) and fluorescence emission spectrum (red) of **4** (80 μM) in H_2_O. (b) UV–vis spectra of **4** (50 μM) in DMEM:DMSO (1:1) over the course of 24 h at 37 °C. (c) ESI mass spectra of **4** (40 μM) in H_2_O:DMSO (125:1) in the presence of glutathione (400 μM) at 37 °C after 0 or 72 h incubation.

The experimentally determined LogP values for **1**-**4**, obtained using the shake-flask method and UV–vis spectroscopy, varied from −0.88 ± 0.01 to 1.50 ± 0.08. The LogP values are directly correlated to the bulkiness of the polypyridyl ligand in **1**-**4** (Table S9). Time course UV–vis spectroscopy and ESI mass spectrometry studies were carried out to determine the stability of **1**-**4** in solutions relevant for cell-based studies. During cell-based studies, stock solutions of test compounds are typically prepared in DMSO prior to dilution in cell culture media. In DMSO, the absorption bands associated with **1**-**4** (50 μM) remained unchanged over the course of 24 h at 37 °C, indicative of good stability (Figure S31). Equally, in cell culture media DMEM:DMSO (200:1 or 1:1), the absorption spectra associated with **1, 2**, and **4** (50 μM) remained largely unaltered over the course of 24 h at 37 °C, suggestive of good stability (Figures 2 and S32). There was a slight reduction (up to 19%) in the intensity of the UV–vis trace associated with **3** in cell culture media, however there was no change in the band wavelengths, indicating some instability but no major structural modifications (Figure S32). The ESI mass spectra of **1**-**4** (40 μM) in H_2_O:DMSO (125:1) with and without ascorbic acid or glutathione (10 equivalence), recorded at 37 °C, displayed characteristic molecular ion peaks with the expected isotopic pattern for **1**-**4** throughout a 72 h incubation period (Figures 2 and S33–S44). This is indicative of good stability in aqueous solution, with and without biologically relevant reducing agents.

The cytotoxicity of **1**-**4** toward bulk breast cancer cells (HMLER), bulk osteosarcoma cells (U2OS), breast CSC-enriched cells (HMLER-shEcad), and osteosarcoma stem cell (OSC)-enriched cells (U2OS-MTX) was determined using the MTT assay. The corresponding IC_50_ values (concentration at which half the cells become unviable) were determined from dose–response curves (Figure S45–S48) and are presented in Table 1. The 2,2′-bipyridine- and 1,10-phenanthroline-bearing complexes **1** and **2** displayed micromolar potencies toward the cell lines tested, whereas the 3,4,7,8-tetramethyl-1,10-phenanthroline- and 4,7-diphenyl-1,10-phenanthroline-bearing complexes **3** and **4** exhibited submicromolar potencies. All of the complexes were significantly more potent (*p* < 0.05, n = 18) toward bulk breast cancer cells than breast CSCs. Notably, **1** and **2** were significantly more potent (*p* < 0.05, n = 18) toward OSCs than bulk osteosarcoma cells. Complex **3** was more toxic toward bulk osteosarcoma cells than OSCs and **4** was equipotent toward the two cell types. The 4,7-diphenyl-1,10-phenanthroline-bearing complex **4** was the most potent across the series, and notably displayed 5- to 121-fold greater activity toward breast CSCs and OSCs than cisplatin (clinically approved anticancer metallodrug) and salinomycin (established anti-CSC agent).^13^ Additionally, **4** was significantly more potent toward breast CSCs than a recently reported cyclometalated half-sandwich rhodium­(III) complex, and **4** displayed comparable or lower potency toward OSCs than gallium­(III)-polypyridyl complexes with and without nonsteroidal anti-inflammatory drugs.^8,13a,14^

**Table 1:** IC_50_ Values of Cyclometalated Rhodium­(III)-Polypyridyl Complexes **1**-**4**, Cisplatin, and Salinomycin against HMLER, HMLER-shEcad, U2OS, and U2OS-MTX Cells

Compound	HMLER IC_50_ [μM]^a^	HMLER-shEcad IC_50_ [μM]^a^	U2OS IC_50_ [μM]^a^	U2OS-MTX IC_50_ [μM]^a^
**1**	5.50 ± 0.38	16.91 ± 0.22	24.33 ± 0.41	13.53 ± 0.22
**2**	9.35 ± 0.03	15.54 ± 0.75	26.14 ± 0.39	14.40 ± 0.51
**3**	0.23 ± 0.01	0.36 ± 0.02	0.45 ± 0.04	0.76 ± 0.01
**4**	0.08 ± 0.01	0.27 ± 0.01	0.26 ± 0.01	0.28 ± 0.02
cisplatin^b^	2.57 ± 0.02	5.65 ± 0.30	16.30 ± 0.50	33.87 ± 3.71
salinomycin^b^	11.43 ± 0.42	4.23 ± 0.35	6.09 ± 1.06	1.49 ± 0.26

a Determined after 72 h incubation (mean of three independent experiments ± SD).

b Reported in ref 13.

The ability of **1**-**4** to disrupt the formation and viability of three-dimensional breast CSC-enriched mammospheres and OSC-enriched sarcospheres was evaluated. All of the rhodium­(III) complexes were able to inhibit the formation of mammospheres or sarcospheres when dosed at sub-lethal concentrations (IC_20_ value for 5 or 10 days) (Figure 3a-b). Similar outcomes were observed upon treatment of mammospheres and sarcospheres with cisplatin or salinomycin under identical conditions (Figure 3a-b). In terms of mammosphere or sarcosphere viability, the potency of **1**-**4** generally increased according to the bulkiness of the polypyridyl ligand present, with the 4,7-diphenyl-1,10-phenanthroline-bearing complex **4** displaying low micromolar IC_50_ values (Table 2 and Figures S49–S50). Markedly, the potency of **4** toward mammospheres and sarcospheres was 4- to 19-fold greater than cisplatin and salinomycin.^13c,15^

**Table 2:** IC_50_ Values of Cyclometalated Rhodium­(III)-Polypyridyl Complexes **1**-**4**, Cisplatin, and Salinomycin against HMLER-shEcad Mammospheres and U2OS-MTX Sarcospheres

Compound	Mammospheres IC_50_ [μM]^a^	Sarcospheres IC_50_ [μM]^a^
**1**	>100	4.94 ± 0.05
**2**	51.26 ± 1.50	17.06 ± 0.28
**3**	3.91 ± 0.51	3.56 ± 0.92
**4**	0.98 ± 0.21	1.12 ± 0.01
cisplatin^b^	13.50 ± 2.34	16.49 ± 0.20
salinomycin^b^	18.50 ± 0.50	4.70 ± 0.08

a Determined after 5 or 10 days incubation (mean of three independent experiments ± SD).

b Reported in references 13c and 14.

**Figure 3:**
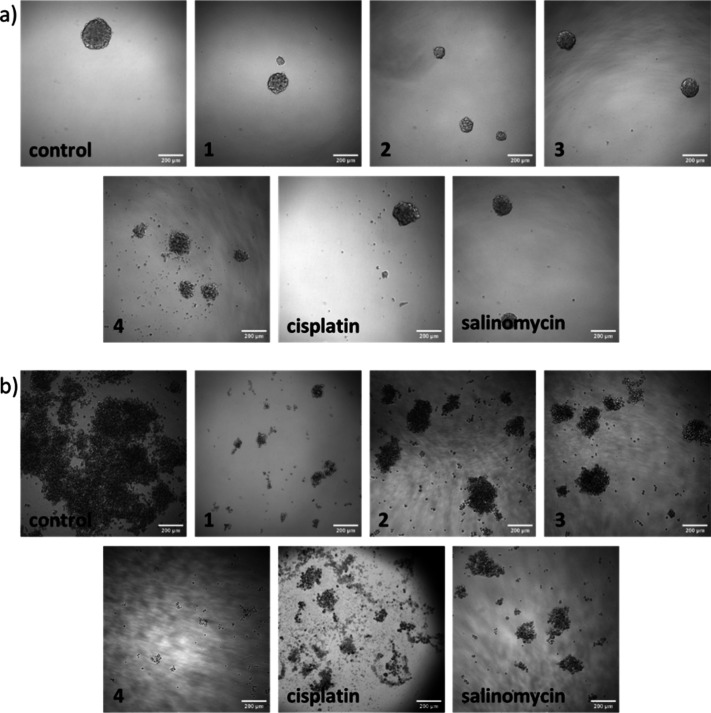
(a) Representative bright-field images (× 10) of mammospheres in the absence and presence of rhodium­(III)-polypyridyl complexes **1**-**4**, cisplatin or salinomycin (IC_20_ for 5 days). Scale bar = 200 μm. (b) Representative bright-field images (× 10) of sarcospheres in the absence and presence of rhodium­(III)-polypyridyl complexes **1**-**4**, cisplatin or salinomycin (IC_20_ for 10 days). Scale bar = 200 μm.

In conclusion, our results demonstrate that cyclometalated rhodium­(III)-polypyridyl complexes **1**-**4** exhibit excellent stability in biologically relevant solutions and strong *in vitro* activity toward both monolayer and three-dimensionally cultured breast CSCs and OSCs, with potencies comparable to or exceeding those of cisplatin and salinomycin. These findings provide strong impetus for the further development of rhodium complexes as anti-CSC agents and warrant detailed molecular and biological investigations to elucidate their underlying anti-CSC mechanisms of action.

## Supplementary Material



## Notes

Details of synthetic methods, X-ray crystallography data, graphical NMR, ESI, and IR spectra, biophysical data and *in vitro* data (PDF)
