# Supplementary material for: Cyclometalated Rhodium(III) Polypyridyl Complexes with Anti-Cancer Stem Cell Activity
Source: Organometallics. 2026 Mar 12;45(6):628–32. doi: 10.1021/acs.organomet.6c00018 (PMC13014534; doi:10.1021/acs.organomet.6c00018)
Supplement: Supplementary file 1 [file om6c00018_si_001.pdf]

Supporting Information for

Cyclometalated Rhodium(III) Polypyridyl Complexes with Anti-Cancer Stem Cell Activity

Hao Ren, Kuldip Singh, and Kogularamanan Suntharalingam*

School of Chemistry, University of Leicester, Leicester, LE1 7RH, UK
* To whom correspondence should be addressed:
Email: k.suntharalingam@leicester.ac.uk

<u>Table of Content</u>

Experimental Details
Figure S1. ^{1}H NMR spectrum of **1** in DMSO-d_6.
Figure S2. $^{13}C\{^{1}H\}$ NMR spectrum of **1** in DMSO-d_6.
Figure S3. $^{19}F\{^{1}H\}$ NMR spectrum of **1** in DMSO-d_6.
Figure S4. $^{31}P\{^{1}H\}$ NMR spectrum of **1** in DMSO-d_6.
Figure S5. ^{1}H NMR spectrum of **2** in DMSO-d_6.
Figure S6. $^{13}C\{^{1}H\}$ NMR spectrum of **2** in DMSO-d_6.
Figure S7. $^{19}F\{^{1}H\}$ NMR spectrum of **2** in DMSO-d_6.
Figure S8. $^{31}P\{^{1}H\}$ NMR spectrum of **2** in DMSO-d_6.
Figure S9. ^{1}H NMR spectrum of **3** in DMSO-d_6.
Figure S10. $^{13}C\{^{1}H\}$ NMR spectrum of **3** in DMSO-d_6.
Figure S11. $^{19}F\{^{1}H\}$ NMR spectrum of **3** in DMSO-d_6.
Figure S12. $^{31}P\{^{1}H\}$ NMR spectrum of **3** in DMSO-d_6.
Figure S13. ^{1}H NMR spectrum of **4** in DMSO-d_6.
Figure S14. $^{13}C\{^{1}H\}$ NMR spectrum of **4** in DMSO-d_6.
Figure S15. $^{19}F\{^{1}H\}$ NMR spectrum of **4** in DMSO-d_6.
Figure S16. $^{31}P\{^{1}H\}$ NMR spectrum of **4** in DMSO-d_6.
Figure S17. ATR-FTIR spectrum of **1** in the solid form.
Figure S18. ATR-FTIR spectrum of **2** in the solid form.
Figure S19. ATR-FTIR spectrum of **3** in the solid form.
Figure S20. ATR-FTIR spectrum of **4** in the solid form.
Figure S21. (Top) Theoretical isotope model for [**1**-PF_6]$^+$ ($C_{27}H_{21}ClN_4Rh$) and (bottom) the experimentally determined high-resolution ESI-TOF mass spectrum for complex **1**.
Figure S22. (Top) Theoretical isotope model for [**2**-PF_6]$^+$ ($C_{29}H_{21}ClN_4Rh$) and (bottom) the experimentally determined high-resolution ESI-TOF mass spectrum for complex **2**.

Figure S23. (Top) Theoretical isotope model for [**3**-PF_6]$^+$ ($C_{33}H_{29}ClN_4Rh$) and (bottom) the experimentally determined high-resolution ESI-TOF mass spectrum for complex **3**.
Figure S24. (Top) Theoretical isotope model for [**4**-PF_6]$^+$ ($C_{41}H_{29}ClN_4Rh$) and (bottom) the experimentally determined high-resolution ESI-TOF mass spectrum for complex **4**.
Table S1. Selected crystallographic data for complexes **1** and **2**.
Table S2. Selected crystallographic data for complexes **3** and **4**.
Table S3. Selected bond lengths (Å) and angles (°) for complex **1**.
Table S4. Selected bond lengths (Å) and angles (°) for complex **2**.

Table S5. Selected bond lengths (Å) and angles (°) for complex **3**.
Table S6. Selected bond lengths (Å) and angles (°) for complex **4**.
Table S7. Absorbance, emission (λ_{ex} = 340 nm), and quantum yield data for **1**–**4** (80 µM) in acetonitrile.
Table S8. Absorbance, emission (λ_{ex} = 340 nm), and quantum yield data for **1**–**4** (80 µM) in H_2O.
Figure S25. UV-vis spectra of (a) **1**, (b) **2**, (c) **3**, and (d) **4** (all 80 µM) in MeCN.
Figure S26. Fluorescence emission spectra of (a) **1**, (b) **2**, (c) **3**, and (d) **4** (all 80 µM) in MeCN (λ_{ex} = 340 nm).
Figure S27. UV-vis spectra of (a) **1**, (b) **2**, (c) **3**, and (d) **4** (all 80 µM) in H_2O.
Figure S28. Fluorescence emission spectra of (a) **1**, (b) **2**, (c) **3**, and (d) **4** (all 80 µM) in H_2O (λ_{ex} = 340 nm).
Figure S29. Fluorescence emission area versus absorption of **1-4** and [Ru(2,2'-bipyridine)$_3$]Cl_2 in MeCN (λ_{ex} = 340 nm). Used to calculate the quantum yield for **1-4**.
Figure S30. Fluorescence emission area versus absorption of **1-4** and [Ru(2,2'-bipyridine)$_3$]Cl_2 in H_2O (λ_{ex} = 340 nm). Used to calculate the quantum yield for **1-4**.
Table S9. Experimentally determined LogP values for **1-4**.
Figure S31. UV-vis spectra of (a) **1**, (b) **2**, (c) **3** or (d) **4** (all 50 µM) in DMSO over the course of 24 h at 37 °C.
Figure S32. UV-vis spectra of (a) **1**, (b) **2**, (c) **3** or (d) **4** (all 50 µM) in DMEM:DMSO (200:1 or 1:1) over the course of 24 h at 37 °C.
Figure S33. ESI mass spectra of **1** (40 µM) in H_2O:DMSO (125:1) at 37 °C after (a) 0 h, (b) 24 h, (c) 48 h or (d) 72 h incubation.
Figure S34. ESI mass spectra of **2** (40 µM) in H_2O:DMSO (125:1) at 37 °C after (a) 0 h, (b) 24 h, (c) 48 h or (d) 72 h incubation.
Figure S35. ESI mass spectra of **3** (40 µM) in H_2O:DMSO (125:1) at 37 °C after (a) 0 h, (b) 24 h, (c) 48 h or (d) 72 h incubation.
Figure S36. ESI mass spectra of **4** (40 µM) in H_2O:DMSO (125:1) at 37 °C after (a) 0 h, (b) 24 h, (c) 48 h or (d) 72 h incubation.
Figure S37. ESI mass spectra of **1** (40 µM) in H_2O:DMSO (125:1) in the presence of ascorbic acid (400 µM) at 37 °C after (a) 0 h, (b) 24 h, (c) 48 h or (d) 72 h incubation.
Figure S38. ESI mass spectra of **2** (40 µM) in H_2O:DMSO (125:1) in the presence of ascorbic acid (400 µM) at 37 °C after (a) 0 h, (b) 24 h, (c) 48 h or (d) 72 h incubation.

Figure S39. ESI mass spectra of **3** (40 µM) in H_2O:DMSO (125:1) in the presence of ascorbic acid (400 µM) at 37 °C after (a) 0 h, (b) 24 h, (c) 48 h or (d) 72 h incubation.

Figure S40. ESI mass spectra of **4** (40 µM) in H_2O:DMSO (125:1) in the presence of ascorbic acid (400 µM) at 37 °C after (a) 0 h, (b) 24 h, (c) 48 h or (d) 72 h incubation.

Figure S41. ESI mass spectra of **1** (40 µM) in H_2O:DMSO (125:1) in the presence of glutathione (400 µM) at 37 °C after (a) 0 h, (b) 24 h, (c) 48 h or (d) 72 h incubation.

Figure S42. ESI mass spectra of **2** (40 µM) in H_2O:DMSO (125:1) in the presence of glutathione (400 µM) at 37 °C after (a) 0 h, (b) 24 h, (c) 48 h or (d) 72 h incubation.

Figure S43. ESI mass spectra of **3** (40 µM) in H_2O:DMSO (125:1) in the presence of glutathione (400 µM) at 37 °C after (a) 0 h, (b) 24 h, (c) 48 h or (d) 72 h incubation.

Figure S44. ESI mass spectra of **4** (40 µM) in H_2O:DMSO (125:1) in the presence of glutathione (400 µM) at 37 °C after (a) 0 h, (b) 24 h, (c) 48 h or (d) 72 h incubation.

Figure S45. Representative dose-response curves for the treatment of HMLER cells with (a) **1**, (b) **2**, (c) **3** or (d) **4** after 72 h incubation.

Figure S46. Representative dose-response curves for the treatment of HMLER-shEcad cells with (a) **1**, (b) **2**, (c) **3** or (d) **4** after 72 h incubation.

Figure S47. Representative dose-response curves for the treatment of U2OS cells with (a) **1**, (b) **2**, (c) **3** or (d) **4** after 72 h incubation.

Figure S48. Representative dose-response curves for the treatment of USOS-MTX cells with (a) **1**, (b) **2**, (c) **3** or (d) **4** after 72 h incubation.

Figure S49. Representative dose-response curves for the treatment of HMLER-shEcad mammospheres with **1-4** after 5 days incubation.

Figure S50. Representative dose-response curves for the treatment of U2OS-MTX sarcospheres with **1-4** after 10 days incubation.

References

Experimental Details

Materials and Methods. All synthetic procedures were performed under normal atmospheric conditions except specific reactions. ^{1}H, $^{13}C\{^{1}H\}$, $^{31}P\{^{1}H\}$ and $^{19}F\{^{1}H\}$ NMR were recorded at room temperature on a Bruker Avance 400 spectrometer (^{1}H 400.0 MHz, ^{13}C 150.9 MHz, ^{31}P 162.0 MHz, ^{19}F 376.5 MHz) with chemical shifts (δ, ppm) reported relative to the solvent peaks of the deuterated solvent. Fourier transform infrared (FTIR) spectra were recorded with an IRAffinity-1S Shimadzu spectrophotometer. UV-vis absorption spectra were recorded on a Cary 3500 UV-vis spectrophotometer. Elemental analysis of the compounds prepared was performed commercially by the University of Cambridge. $RhCl_3{\cdot}3H_2O$, sodium hexafluorophosphate and all the polypyridyl ligands were purchased from Sigma-Aldrich and used without further purification. 2,2′-(Phenylmethylene)dipyridine (**L^1**) was prepared using previously reported protocols.[1] Solvents were purchased from Fisher and used without further purification.

Synthesis of [Rh(L^1)(2,2'-bipyridine)Cl]PF$_6$ (1). 2,2′-(Phenylmethylene)dipyridine (103 mg, 0.42 mmol) in 2-methoxyethanol (36 mL) and $RhCl_3{\cdot}3H_2O$ (124 mg, 0.42 mmol) in H_2O (12 mL) were stirred under nitrogen at 130 °C for 48 h. 2,2'-Bipyridine (71 mg, 0.45 mmol) in 2-methoxyethanol (2 mL) was then added and the reaction was continued for a further 24 h. The resultant solution was filtered and the filtrated was evaporated to dryness to yield a red oil. The red oil was suspended in DMSO (1 ml) to which was add a saturated aqueous solution of sodium hexafluorophosphate (250 mg in 1 mL). The resulting precipitate was separated by filtration and washed with water (10 mL), diethyl ether (10 mL), and dichloromethane (10 mL) to yield **1** as an orange solid (79.6 mg, 58%). ^{1}H NMR (400 MHz, DMSO-d$_6$): δ 9.05 (d, 1H), 9.00 (d, 1H), 8.93 (d, 1H), 8.56 (t, 2H), 8.48 (td, 1H), 8.42 (td, 1H), 8.18 (td, 1H), 8.06 (td, 2H), 8.00 (td, 1H), 7.85 (td, 1H), 7.81 (dd, 1H), 7.72 (t, 1H), 7.63 (t, 1H), 7.49 (dd, 1H), 7.33 (d, 1H), 7.22 (td, 1H), 7.16 (td, 1H), 7.05 (t, 1H), 6.53 (s, 1H); ^{13}C NMR (150.9 MHz, DMSO-d$_6$): δ 157.81, 157.35, 157.14, 156.76, 154.54, 153.95, 152.95, 151.57, 149.03, 140.68, 140.62, 140.41, 140.39, 139.18, 138.11, 127.47, 127.33, 127.05, 125.45, 125.42, 125.37, 124.86, 124.82, 124.62, 124.58, 124.38, 62.98; ^{31}P NMR (162 MHz, DMSO-d$_6$) δ -144.19 (sept, PF$_6$); ^{19}F NMR (376.5 MHz, DMSO-d$_6$) δ -70.15 (d, PF$_6$); ATR-FTIR (solid, cm^{-1}): 1601.7, 1572.8, 1472.7, 1446.4, 1399.1, 1351.7, 1159.5, 1125.3, 1106.9, 1067.4, 875.3, 830.5, 759.5, 730.5, 630.5, 612.1, 554.2, 493.6, 470.0, 419.9; ESI-MS Calcd. for $C_{27}H_{21}ClN_4Rh$ [M-PF$_6$]$^+$: 539.0510 a.m.u. Found [M-PF$_6$]$^+$: 539.0508 a.m.u.; Anal. Calcd. for $C_{27}H_{21}ClN_4RhPF_6$: C, 47.36; H, 3.09; N, 8.18. Found: C, 47.08; H, 3.01; N, 8.07.

Synthesis of [Rh(L^1)(1,10-phenanthroline)Cl]PF$_6$ (2). 2,2′-(Phenylmethylene)dipyridine (103 mg, 0.42 mmol) in 2-methoxyethanol (18 mL) and $RhCl_3{\cdot}3H_2O$ (124 mg, 0.42 mmol) in H_2O (6 mL) were stirred under nitrogen at 130 °C for 48 h. 1,10-Phenanthroline (83 mg, 0.46 mmol) in 2-methoxyethanol (2 mL) was then added and the reaction was continued for a further 24 h. The resultant solution was filtered and the filtrated was evaporated to dryness to yield a red oil. The red oil was suspended in DMSO (1 ml) to which was add a saturated aqueous solution of sodium hexafluorophosphate (250 mg in 1 mL). The resulting precipitate was separated by filtration and washed with water (10 mL) and diethyl ether (10 mL) to yield **2** as an orange solid (113.0 mg, 80%). ^{1}H NMR (400 MHz, DMSO-d$_6$): δ 9.11-9.09 (m, 2H), 9.05 (d, 1H), 9.00 (t, 2H), 8.52 (q, 2H), 8.25-8.19 (m, 2H), 8.11-8.07 (m, 3H), 7.97-7.90 (m, 2H), 7.69 (td, 1H), 7.54 (dd, 1H), 7.28 (td, 1H), 7.20 (td, 1H), 7.11 (d, 1H), 6.86 (td, 1H), 6.59 (s, 1H); ^{13}C NMR (150.9 MHz, DMSO-d$_6$): δ 157.75, 156.67, 156.57, 154.18, 153.60, 151.51, 149.84, 147.03, 144.72, 140.69, 140.64, 139.74, 139.26, 139.15, 138.18, 131.30, 131.06, 128.38, 127.91, 127.16, 126.22, 125.93, 125.60, 125.29, 124.99, 124.85, 124.68,

124.44, 63.09; ^{31}P NMR (162 MHz, DMSO-d_6) δ -144.20 (sept, PF_6); ^{19}F NMR (376.5 MHz, DMSO-d_6) δ -70.15 (d, PF_6); ATR-FTIR (solid, cm^{-1}): 1611.9, 1519.8, 1476.0, 1452.8, 1427.0, 1347.5, 1142.4, 877.4, 838.7, 774.3, 725.4, 650.7, 627.5, 614.6, 556.2; ESI-MS Calcd. for $C_{29}H_{21}ClN_4Rh$ $[M-PF_6]^+$: 563.0510 a.m.u. Found $[M-PF_6]^+$: 563.0509 a.m.u.; Anal. Calcd. for $C_{29}H_{21}ClN_4RhPF_6·0.75H_2O$: C, 48.22; H, 3.14; N, 7.76. Found: C, 48.57; H, 2.97; N, 7.23.

Synthesis of [Rh(L^1)(3,4,7,8-tetramethyl-1,10-phenanthroline)Cl]PF_6 (3). 2,2′-(Phenylmethylene)dipyridine (130 mg, 0.53 mmol) in 2-methoxyethanol (21 mL) and $RhCl_3·3H_2O$ (158 mg, 0.53 mmol) in H_2O (7 mL) were stirred under nitrogen at 130 °C for 48 h. 3,4,7,8-Tetramethyl-1,10-phenanthroline (137 mg, 0.58 mmol) in 2-methoxyethanol (3 mL) was then added and the reaction was continued for a further 24 h. The resultant solution was filtered and the filtrated was evaporated to dryness to yield a red oil. The red oil was suspended in DMSO (1 ml) to which was add a saturated aqueous solution of sodium hexafluorophosphate (250 mg in 1 mL). The resulting precipitate was dissolved in chloroform (1 mL) and added to cold diethyl ether (20 mL). This produced a precipitate which was collected by filtration and washed with acetonitrile (10 mL) and diethyl ether (10 mL) to yield **3** as an orange solid (53.3 mg, 13%). ^1H NMR (400 MHz, DMSO-d_6): δ 9.08 (d, 1H), 8.71 (s, 2H), 8.61 (q, 2H), 8.21 (t, 1H), 8.08 (t, 2H), 7.96-7.91 (m, 2H), 7.69 (t, 1H), 7.53 (d, 1H), 7.28 (t, 1H), 7.19 (t, 1H), 7.05 (d, 1H), 6.88 (t, 1H), 6.56 (s, 1H), 2.94 (s, 3H), 2.92 (s, 3H), 2.57 (s, 3H), 2.46 (s, 3H); ^{13}C NMR (150.9 MHz, DMSO-d_6): δ 157.79, 157.48, 156.69, 154.16, 153.28, 151.34, 149.96, 147.40, 146.54, 145.69, 143.38, 140.59, 140.45, 139.05, 138.22, 134.46, 133.96, 129.55, 129.26, 126.99, 125.55, 125.24, 124.90, 124.83, 124.59, 124.54, 124.35, 124.09, 63.18, 17.41, 14.89, 14.83; ^{31}P NMR (162 MHz, DMSO-d_6) δ -144.20 (sept, PF_6); ^{19}F NMR (376.5 MHz, DMSO-d_6) δ -70.15 (d, PF_6); ATR-FTIR (solid, cm^{-1}): 1612.7, 1528.6, 1478.1, 1456.4, 1432.4, 1389.1, 872.5, 834.0, 775.7, 761.3, 720.4, 626.0, 614.0, 577.9, 556.2, 498.6, 469.7, 452.9; ESI-MS Calcd. for $C_{33}H_{29}ClN_4Rh$ $[M-PF_6]^+$: 619.1136 a.m.u. Found $[M-PF_6]^+$: 619.1135 a.m.u.; Anal. Calcd. for $C_{33}H_{29}ClN_4RhPF_6$: C, 51.82; H, 3.82; N, 7.32. Found: C, 51.57; H, 3.72; N, 7.26.

Synthesis of [Rh(L^1)(4,7-diphenyl-1,10-phenanthroline)Cl]PF_6 (4). 2,2′-(Phenylmethylene)dipyridine (130 mg, 0.53 mmol) in 2-methoxyethanol (21 mL) and $RhCl_3·3H_2O$ (158 mg, 0.53 mmol) in H_2O (7 mL) were stirred under nitrogen at 130 °C for 48 h. 4,7-Diphenyl-1,10-phenanthroline (193 mg, 0.58 mmol) in 2-methoxyethanol (3 mL) was then added and the reaction was continued for a further 24 h. The resultant solution was filtered and the filtrated was evaporated to dryness to yield a red oil. The red oil was suspended in DMSO (1 ml) to which was add a saturated aqueous solution of sodium hexafluorophosphate (250 mg in 1 mL). The resulting precipitate was separated by filtration and washed with water (10 mL) and diethyl ether (10 mL). The solid was then dissolved in acetonitrile (1 mL) and added to cold diethyl ether (20 mL). This produced a precipitate which was collected by filtration and washed with diethyl ether (10 mL) to yield **4** as an orange solid (61.1 mg, 13%). ^1H NMR (400 MHz, DMSO-d_6): δ 9.15 (d, 1H), 9.07 (d, 1H), 9.04 (d, 1H), 8.36 (q, 2H), 8.25 (td, 1H), 8.18 (d, 1H), 8.12 (d, 2H), 8.08 (d, 1H), 8.02-7.97 (m, 2H), 7.81-7.75 (m, 5H), 7.73-7.68 (m, 6H), 7.57 (dd, 1H), 7.32-7.28 (m, 2H), 7.22 (td, 1H), 6.96 (t, 1H), 6.63 (s, 1H); ^{13}C NMR (150.9 MHz, DMSO-d_6): δ 157.86, 157.23, 157.06, 156.73, 154.16, 153.29, 151.88, 150.65, 150.33, 149.54, 147.87, 145.65, 140.72, 140.67, 139.18, 138.25, 135.55, 135.20, 129.96, 129.88, 129.83, 129.30, 129.25, 128.82, 128.50, 127.19, 126.27, 126.16, 126.13, 125.85, 125.62, 125.33, 124.98, 124.94, 124.71, 124.48, 63.13; ^{31}P NMR (162 MHz, DMSO-d_6) δ -144.20 (sept, PF_6); ^{19}F NMR (376.5 MHz, DMSO-d_6) δ -70.15 (d, PF_6); ATR-FTIR (solid, cm^{-1}): 1606.3, 1596.7, 1563.2, 1520.0, 1476.8,

1448.1, 1419.3, 1392.9, 1230.5, 1020.4, 871.7, 833.4, 766.2, 737.5, 701.5, 629.6, 612.8, 555.0, 468.6, 454.2; ESI-MS calcd. for $C_{41}H_{29}ClN_4Rh$ [M-PF$_6$]$^+$: 715.1136 a.m.u. Found [M-PF$_6$]$^+$: 715.1128 a.m.u.; Anal. Calcd. for $C_{41}H_{29}ClN_4RhPF_6 \cdot H_2O$: C, 56.02; H, 3.55; N, 6.37. Found: C, 56.30; H, 3.27; N, 6.10.

X-ray single crystal diffraction analysis. Single crystals of **1-4** were obtained by slow diffusion of diethyl ether into an acetonitrile or dichloromethane solution of **1-4**. Crystals suitable for X-ray diffraction analysis were selected and mounted on a Bruker D8 Quest diffractometer with a Photon III detector and a microfocus source with Cu–Kα radiation (λ = 1.54178) at 150(2) K. Intensities were integrated from data recorded on 1.0° frames by ω rotation. A multiscan method absorption correction (SADABS) with a beam profile was applied.[2] The structures were solved using SHELXS or SHELXT;[3] the datasets were refined by full-matrix least-squares on reflections with $F^2 \geq 2\sigma(F^2)$ values, with anisotropic displacement parameters for all non-hydrogen atoms, and with constrained riding hydrogen geometries, unless otherwise stated;[4] U_{iso}(H) was set at 1.2 (1.5 for methyl groups) times U_{eq} of the parent atom. SHELX was employed through OLEX2 for structure solution and refinement.[5] The structures have been deposited with the Cambridge Crystallographic Data Centre (CCDC 2518219-2518222). This information can be obtained free of charge from www.ccdc.cam.ac.uk/data_request/cif.

Measurement of water-octanol partition coefficient (LogP). The LogP values for **1-4** were determined using the shake-flask method and UV-vis spectroscopy. The 1-octanol used in this experiment was pre-saturated with water. An aqueous solution of **1-4** (500 μL, 100 μM) was incubated with 1-octanol (500 μL) in a 1.5 mL tube. The tube was shaken at room temperature for 24 h. The two phases were separated by centrifugation and the **1-4** content in each phase was determined by UV-vis spectroscopy.

Cell culture. The human mammary epithelial cell lines, HMLER and HMLER-shEcad were kindly donated by Prof. R. A. Weinberg (Whitehead Institute, MIT). HMLER and HMLER-shEcad cells were maintained in Mammary Epithelial Cell Growth Medium (MEGM) with supplements and growth factors (BPE, hydrocortisone, hEGF, insulin, and gentamicin/amphotericin-B). The U2OS bone osteosarcoma cell line was acquired from American Type Culture Collection (ATCC, Manassas, VA, USA) and cultured in Dulbecco's Modified Eagle's Medium (DMEM) supplemented with 10% fetal bovine serum and 1% penicillin. The cells were grown at 310 K in a humidified atmosphere containing 5% CO_2. To gain access to OSC-enriched cells, a full T75 flask of U2OS cells was treated with methotrexate (300 nM) for 4 days.[6] The cells (labelled U2OS-MTX cells) were then used immediately. All cells were grown at 310 K in a humidified atmosphere containing 5% CO_2.

Cytotoxicity studies: MTT assay. Exponentially growing cells were seeded at a density of approximately 5 × 10^3 cells per well in 96-well flat-bottomed microplates and allowed to attach for 24 h prior to addition of compounds. Various concentrations of the test compounds (0.0004-100 μM) were added and incubated for 72 h at 37 °C (total volume 200 μL). Stock solutions of the compounds were prepared as 10 mM DMSO solutions and diluted using cell media. The final concentration of DMSO in each well was ≤1 %. After 72 h, 20 μL of MTT (4 mg mL^{-1} in PBS) was added to each well and the plates incubated for an additional 4 h at 37 °C. The media/MTT mixture was eliminated and DMSO (100 μL per well) was added to dissolve the formazan precipitates. The optical density was measured at 550 nm using a 96-well multiscanner autoreader. Absorbance values were normalised to (DMSO-containing) control wells and plotted as concentration of compound versus % cell viability. IC_{50} values

were interpolated from the resulting dose dependent curves. The reported IC_{50} values are the average of three independent experiments, each consisting of six replicates per concentration level (n = 18).

Mammosphere formation and viability assay. HMLER-shEcad cells (5×10^3) were plated in ultralow-attachment 96-well plates (Corning) and incubated in MEGM supplemented with B27 (Invitrogen), 20 ng mL^{-1} EGF and 4 μg mL^{-1} heparin (Sigma) for 5 days. Studies were also conducted in the presence of test compounds (0-133 μM). Mammospheres treated with test compounds (at their respective IC_{20} values, 5 days) were imaged using an inverted microscope. The viability of the mammospheres was determined by addition of a resazurin-based reagent, TOX8 (Sigma). After incubation for 16 h, the fluorescence of the solutions was read at 590 nm (λ_{ex} = 560 nm). Viable mammospheres reduce the amount of the oxidised TOX8 form (blue) and concurrently increase the amount of the fluorescent TOX8 intermediate (red), indicating the degree of mammosphere cytotoxicity caused by the test compound. Fluorescence values were normalised to DMSO-containing controls and plotted as concentration of test compound versus % mammosphere viability. IC_{50} values were interpolated from the resulting dose dependent curves. The reported IC_{50} values are the average of three independent experiments, each consisting of two replicates per concentration level (n = 6).

Sarcosphere formation and viability assay. U2OS-MTX cells (2.5×10^4) were plated in ultralow-attachment 96-well plates (Corning) and incubated in DMEM supplemented with N2 (Invitrogen), human EGF (10 ng/mL), and human bFGF (10 ng/mL) for 10 days. Studies were also conducted in the presence of test compounds (0-133 μM). Sarcospheres treated with test compounds (at their respective IC_{20} values, 10 days) were imaged using an inverted microscope. The viability of the sarcospheres was determined by addition of a resazurin-based reagent, TOX8 (Sigma). After incubation for 16 h, the fluorescence of the solutions was read at 590 nm (λ_{ex} = 560 nm). Viable sarcospheres reduce the amount of the oxidized TOX8 form (blue) and concurrently increases the amount of the fluorescent TOX8 intermediate (red), indicating the degree of sarcosphere cytotoxicity caused by the test compound. Fluorescence values were normalised to DMSO-containing controls and plotted as concentration of test compound versus % sarcosphere viability. IC_{50} values were interpolated from the resulting dose dependent curves. The reported IC_{50} values are the average of three independent experiments, each consisting of two replicates per concentration level (overall n = 6).

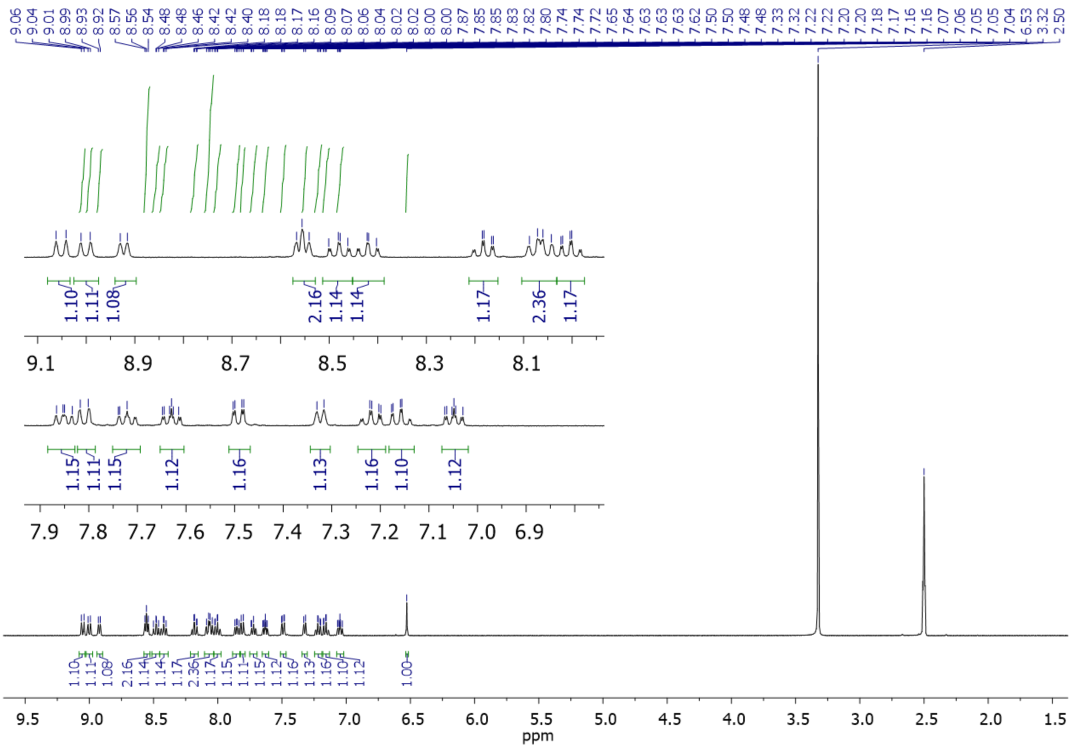

Figure S1. ^{1}H NMR spectrum of **1** in DMSO-d$_6$.

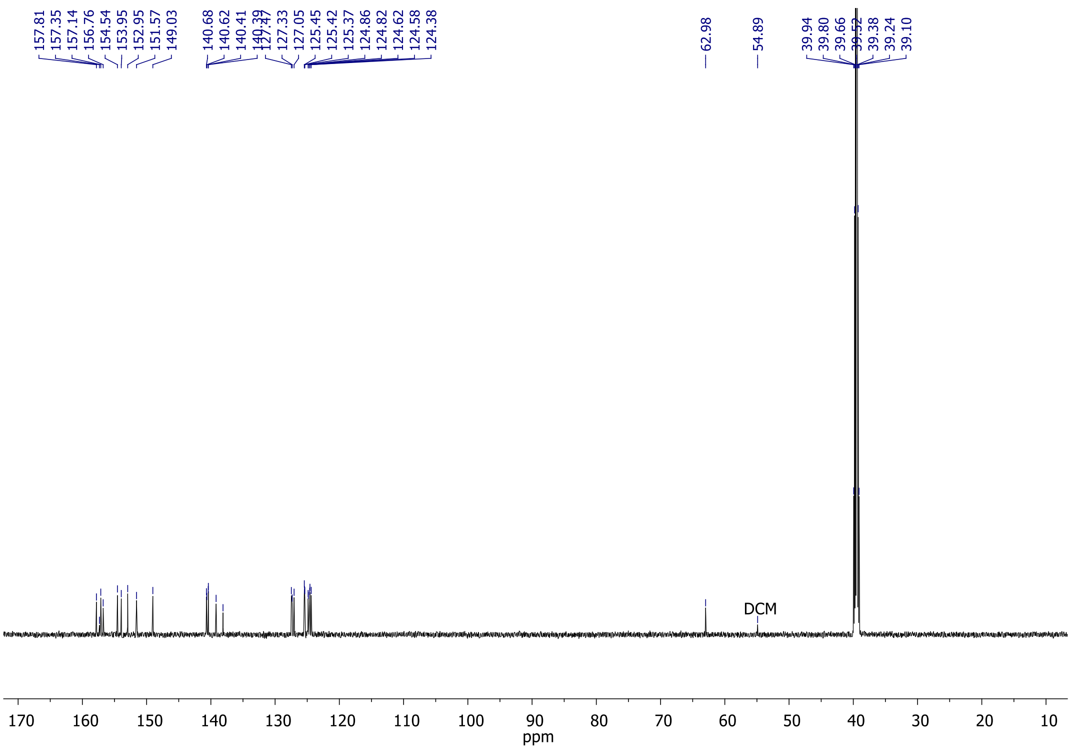

Figure S2. $^{13}C\{^{1}H\}$ NMR spectrum of **1** in DMSO-d$_6$.

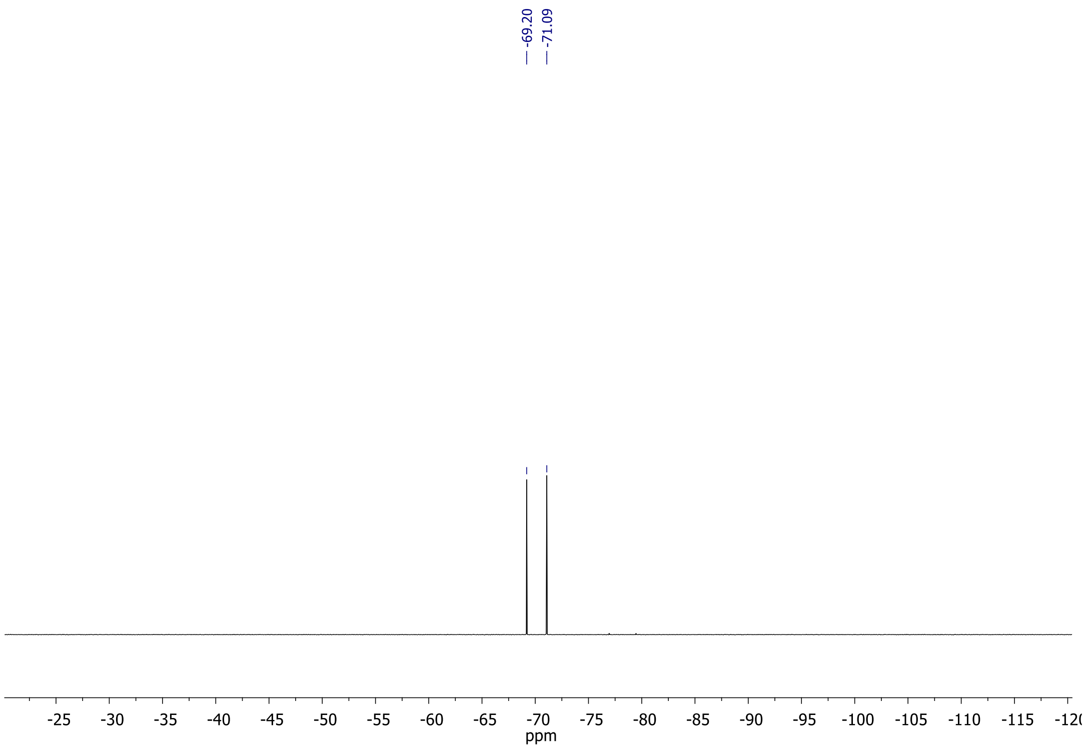

Figure S3. $^{19}F\{^{1}H\}$ NMR spectrum of **1** in DMSO-d_6.

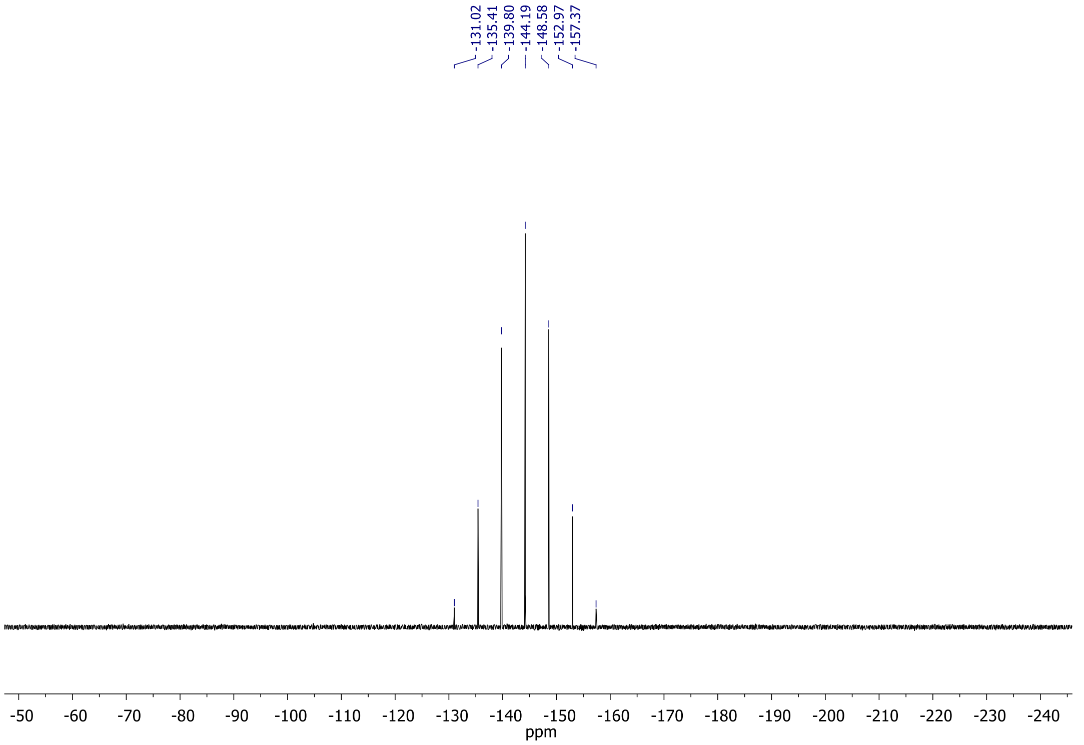

Figure S4. $^{31}P\{^{1}H\}$ NMR spectrum of **1** in DMSO-d_6.

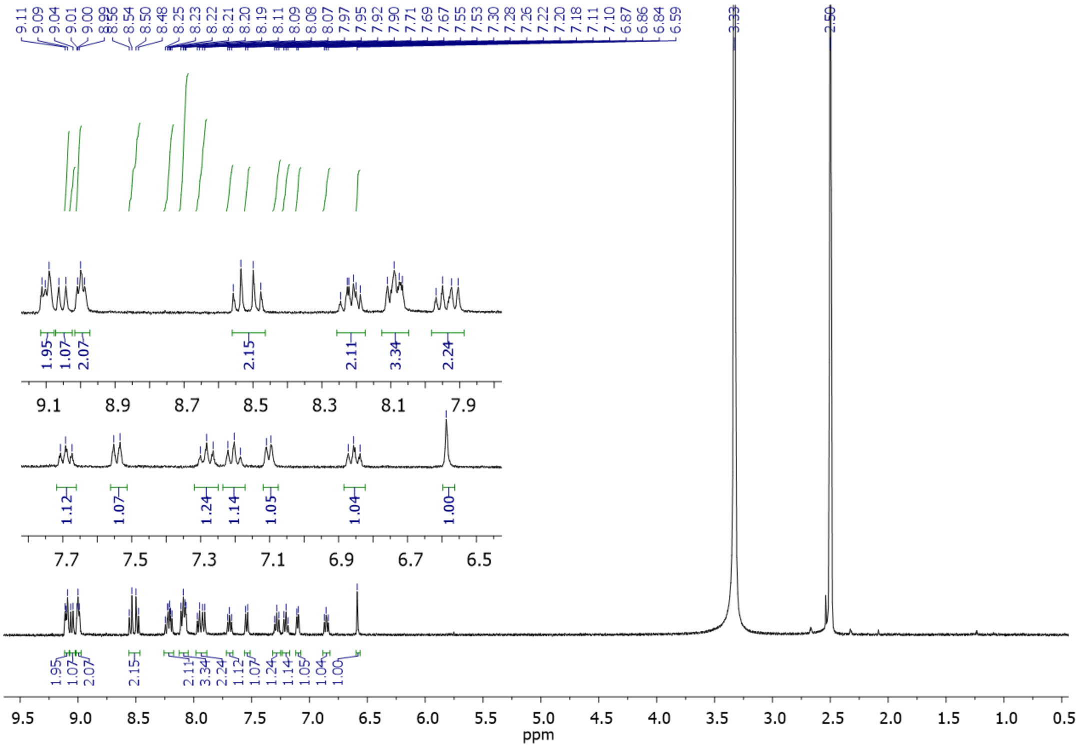

Figure S5. ^{1}H NMR spectrum of **2** in DMSO-d$_6$.

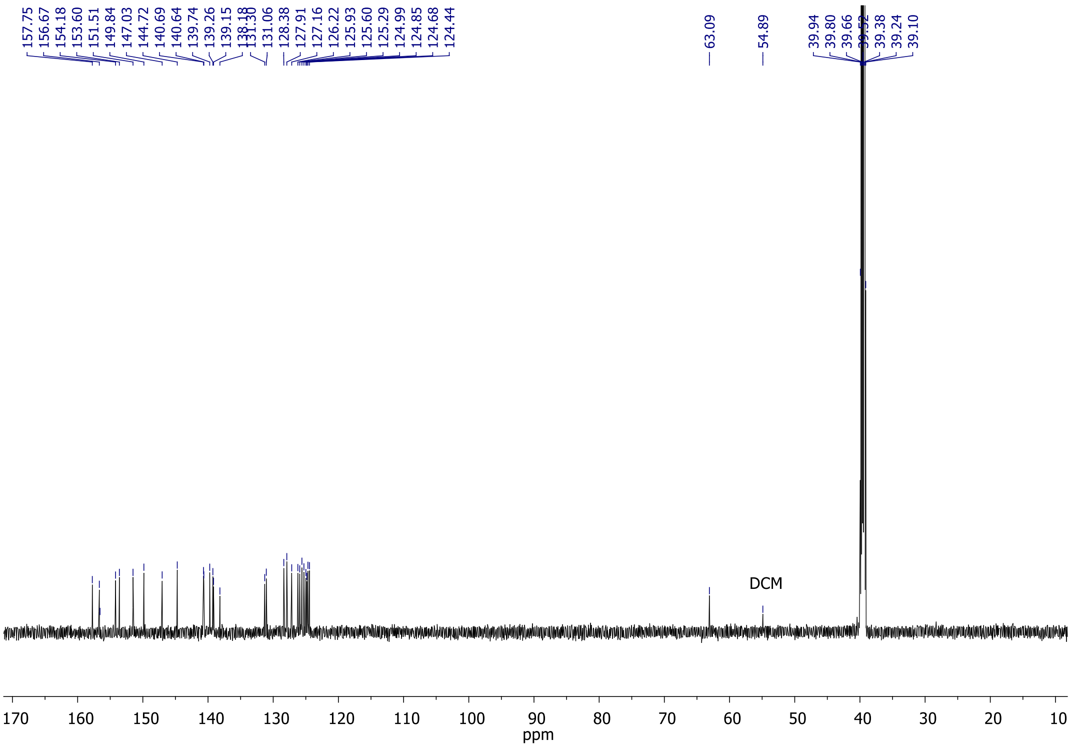

Figure S6. $^{13}C\{^{1}H\}$ NMR spectrum of **2** in DMSO-d$_6$.

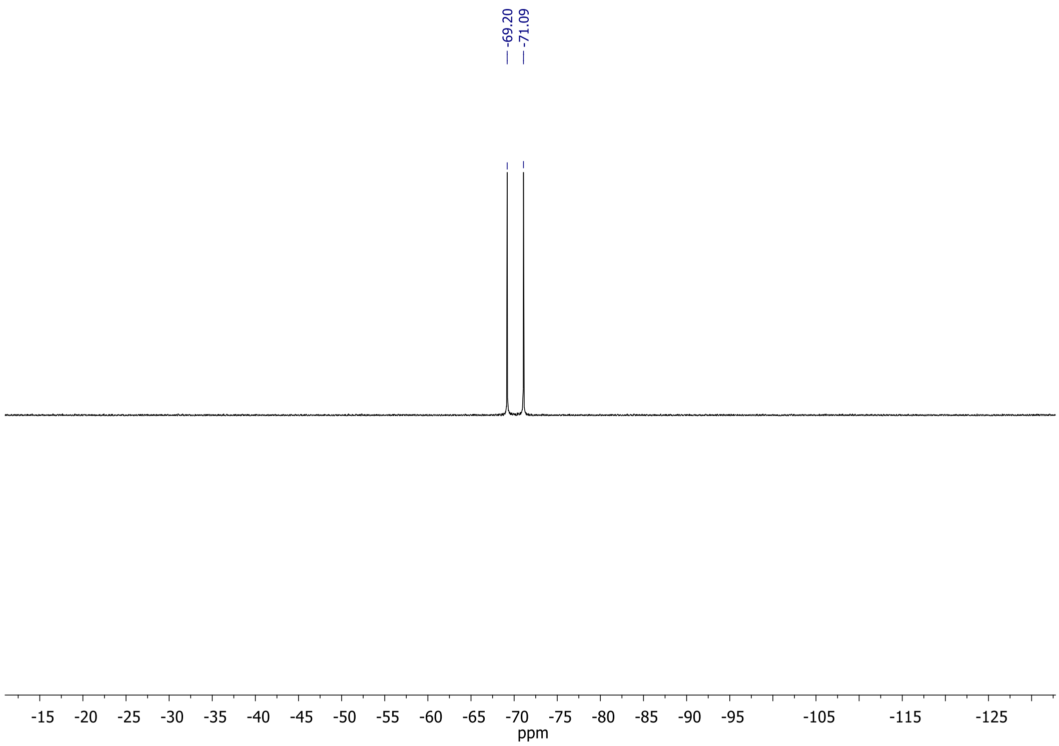

Figure S7. $^{19}F\{^{1}H\}$ NMR spectrum of **2** in DMSO-d_6.

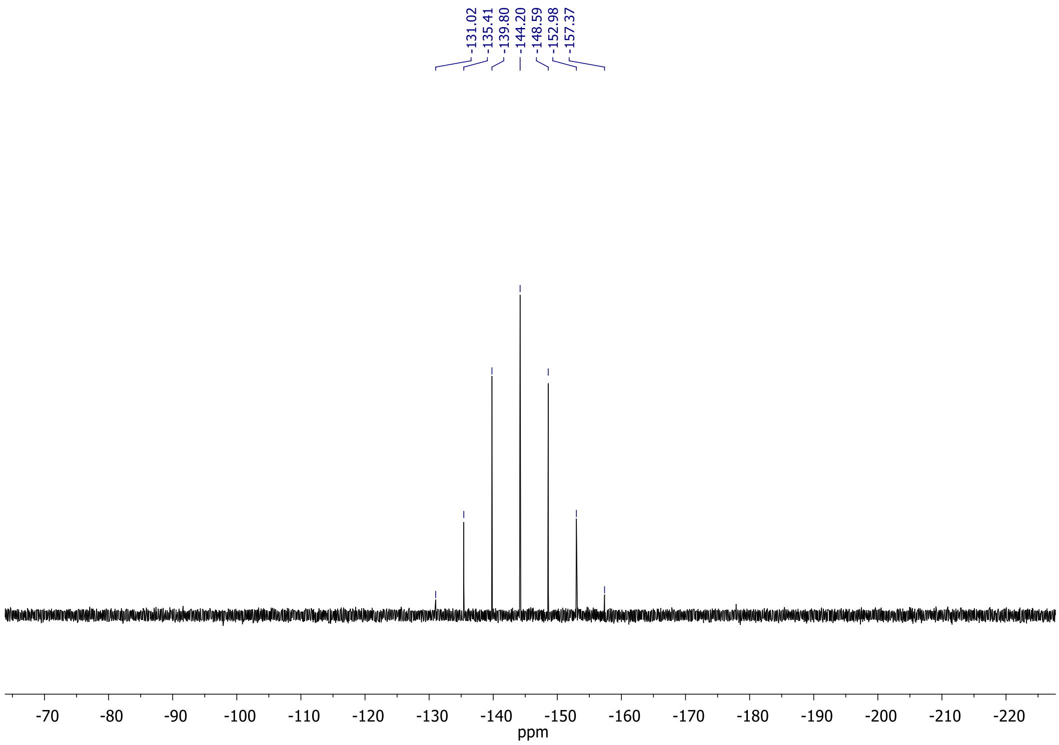

Figure S8. $^{31}P\{^{1}H\}$ NMR spectrum of **2** in DMSO-d_6.

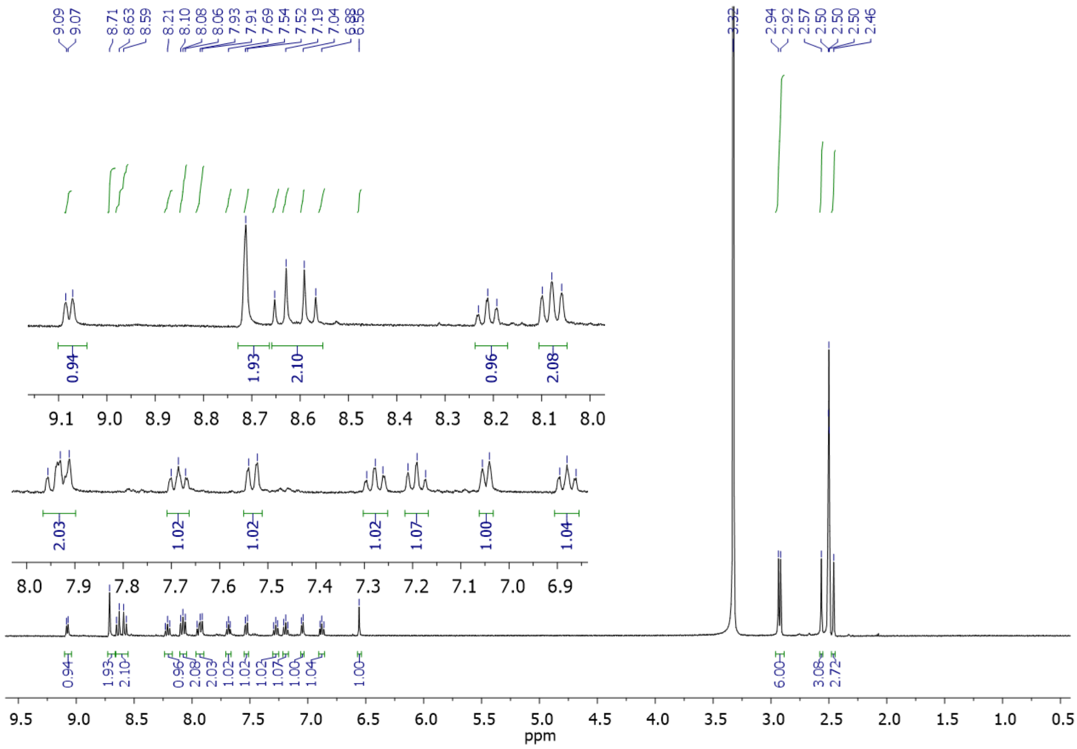

Figure S9. ^{1}H NMR spectrum of **3** in DMSO-d_6.

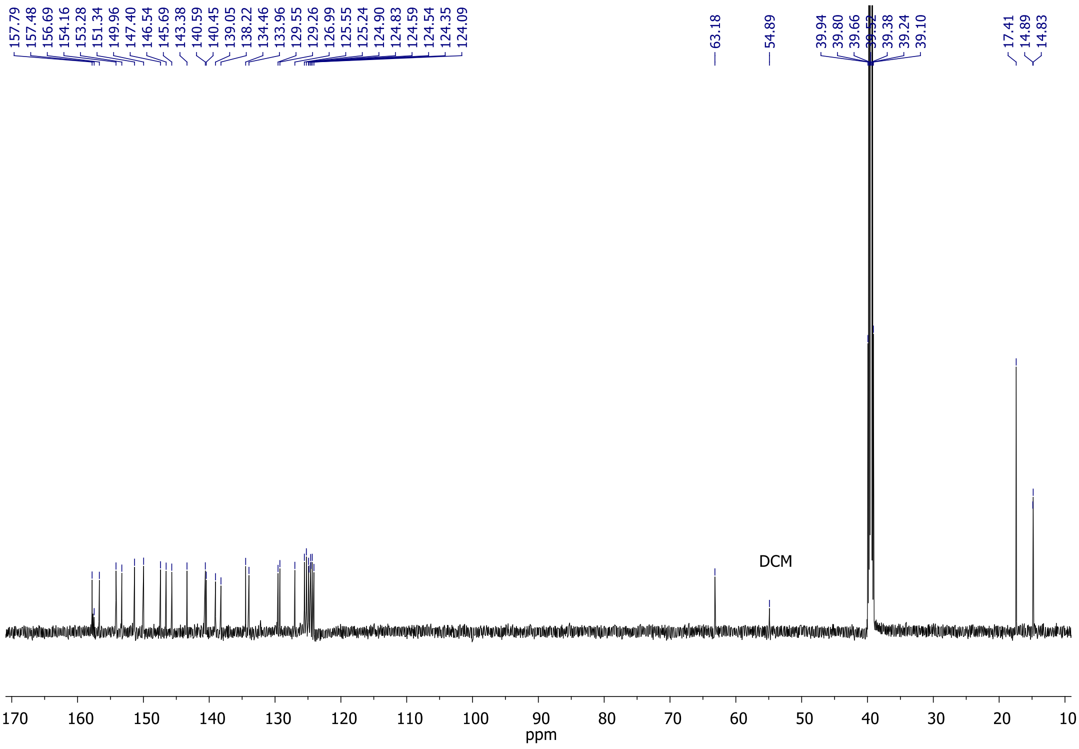

Figure S10. $^{13}C\{^{1}H\}$ NMR spectrum of **3** in DMSO-d_6.

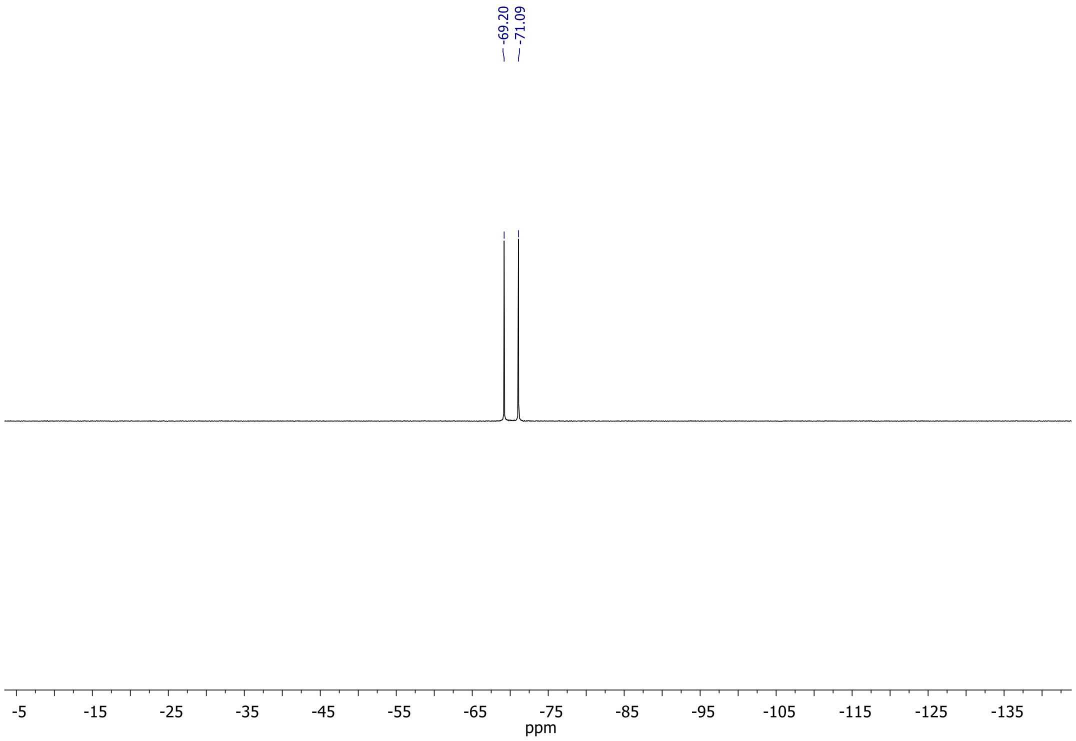

Figure S11. $^{19}F\{^{1}H\}$ NMR spectrum of **3** in DMSO-d6.

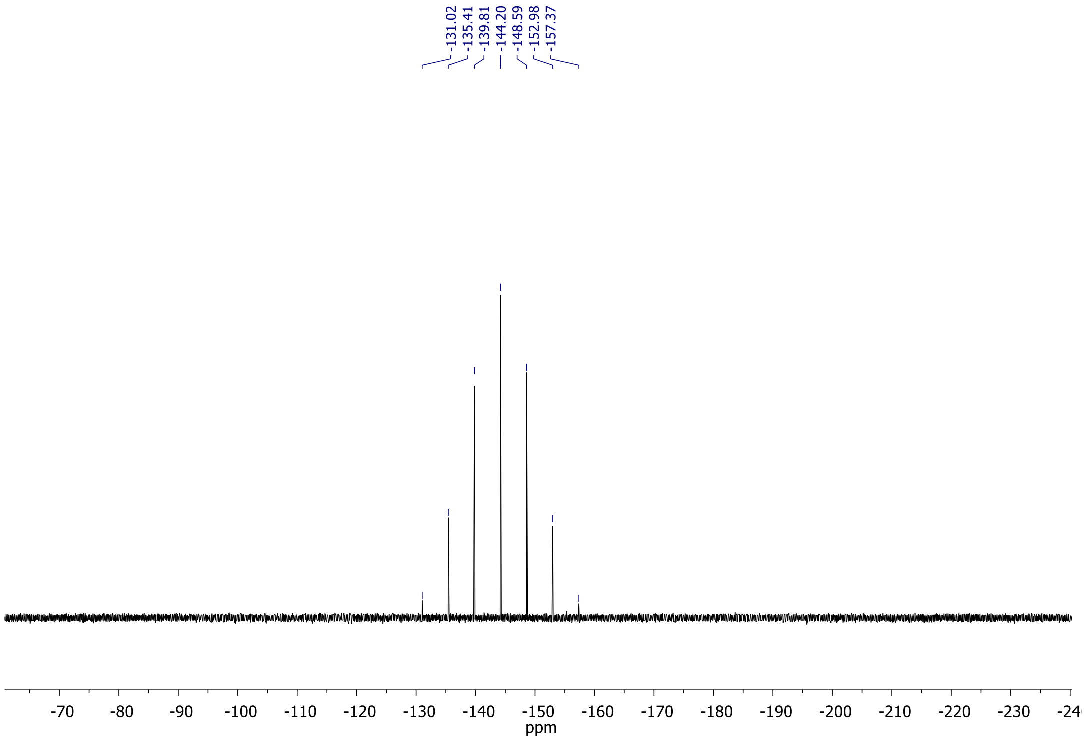

Figure S12. $^{31}P\{^{1}H\}$ NMR spectrum of **3** in DMSO-d6.

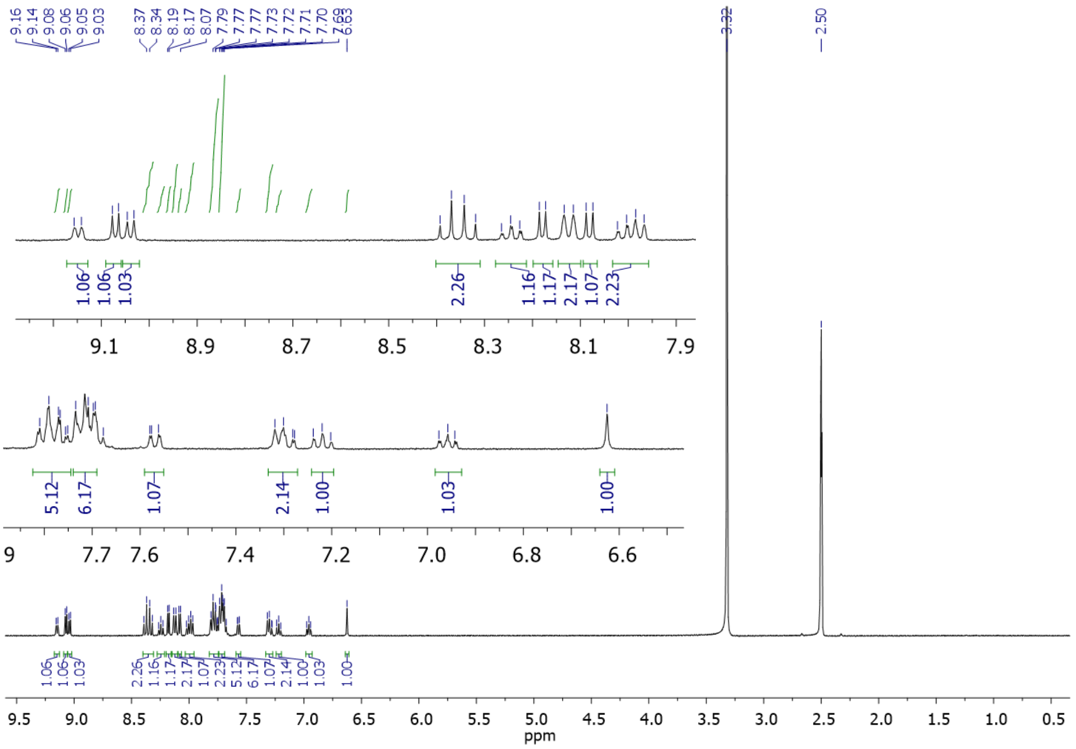

Figure S13. ^{1}H NMR spectrum of **4** in DMSO-d_6.

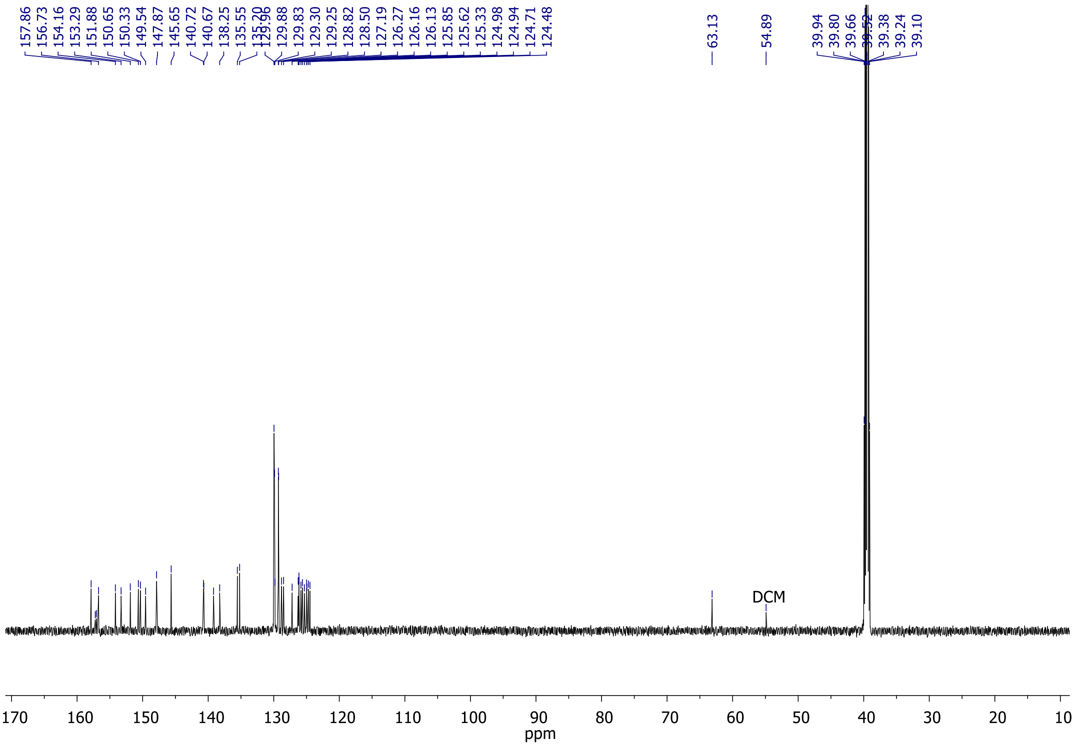

Figure S14. ^{13}C{^{1}H} NMR spectrum of **4** in DMSO-d_6.

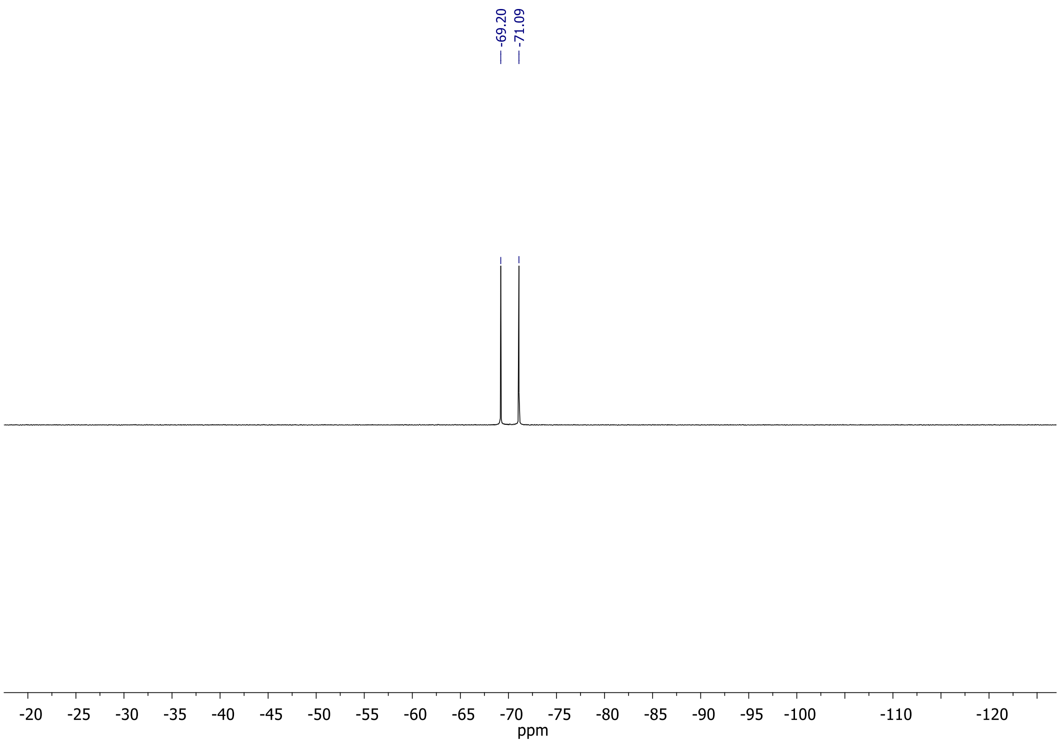

Figure S15. $^{19}F\{^{1}H\}$ NMR spectrum of **4** in DMSO-d$_6$.

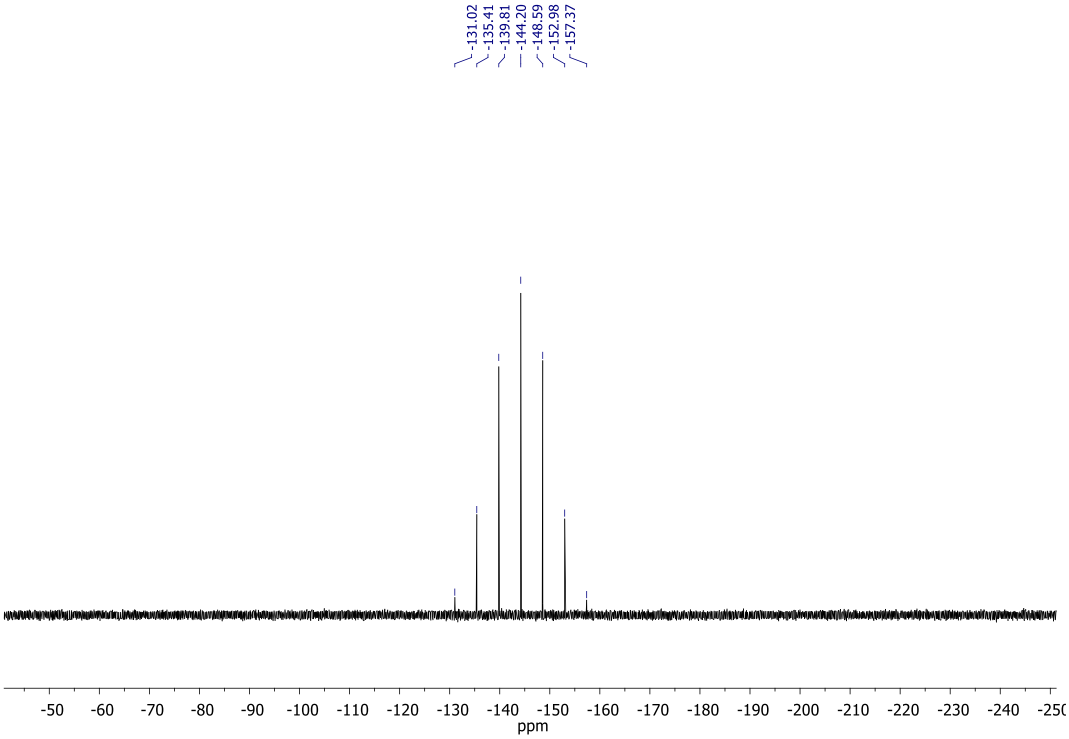

Figure S16. $^{31}P\{^{1}H\}$ NMR spectrum of **4** in DMSO-d$_6$.

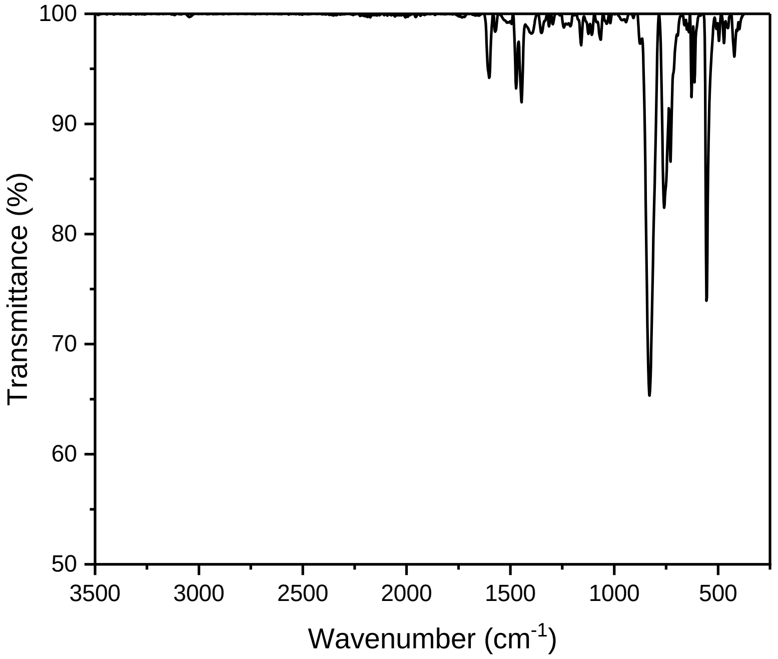

Figure S17. ATR-FTIR spectrum of **1** in the solid form.

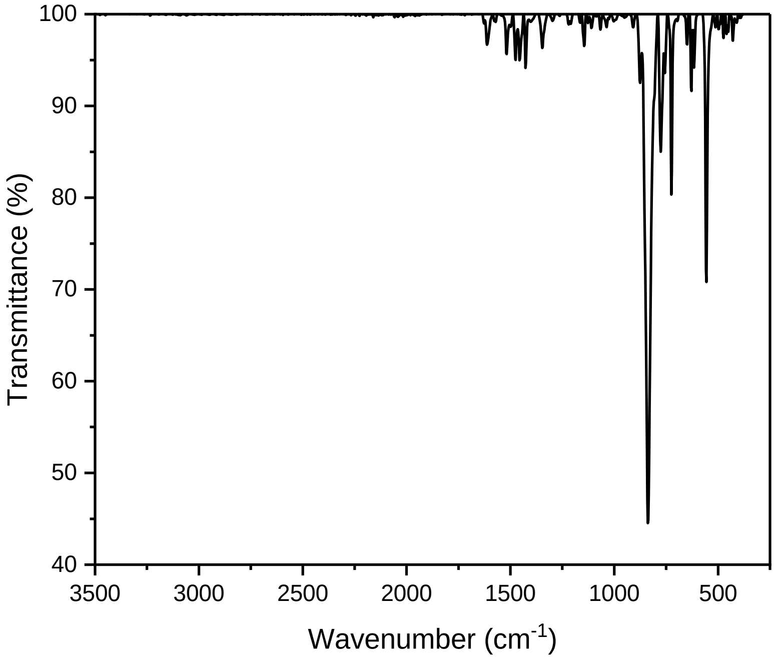

Figure S18. ATR-FTIR spectrum of **2** in the solid form.

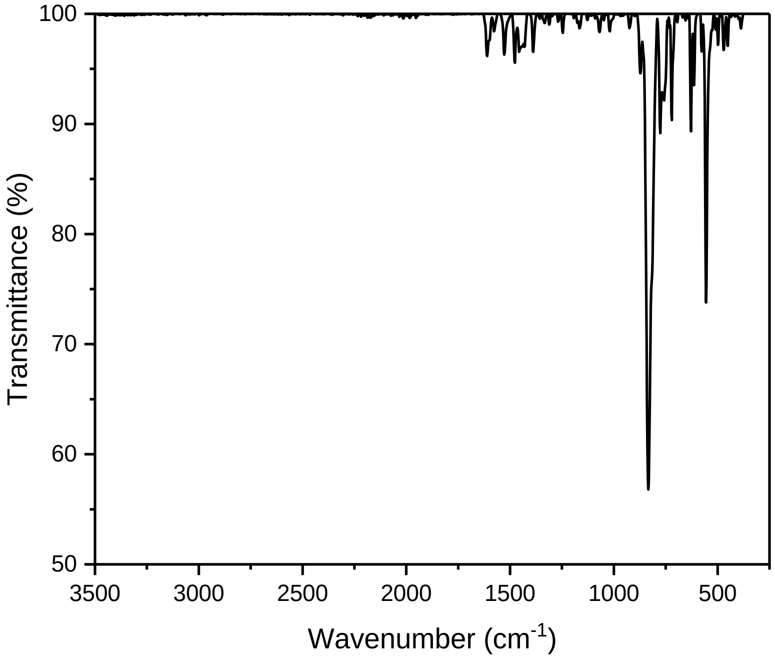

Figure S19. ATR-FTIR spectrum of **3** in the solid form.

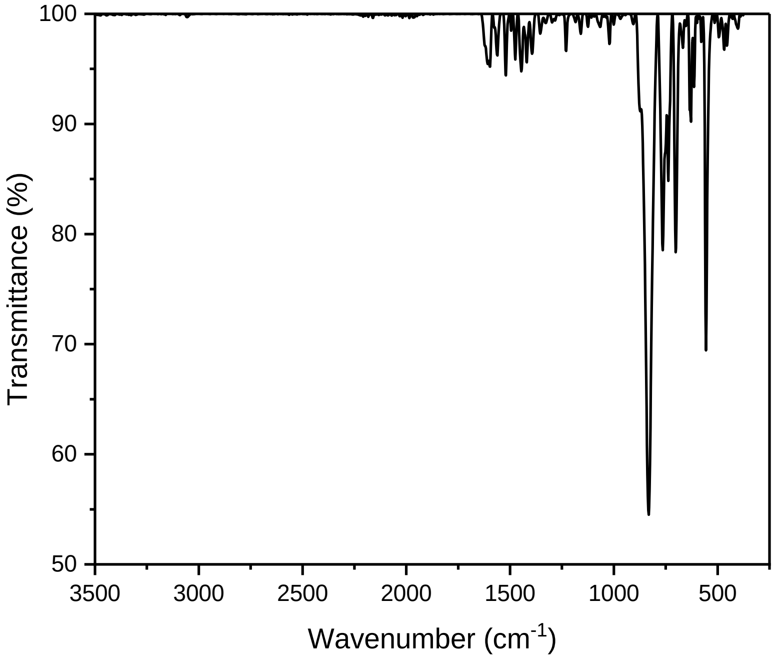

Figure S20. ATR-FTIR spectrum of **4** in the solid form.

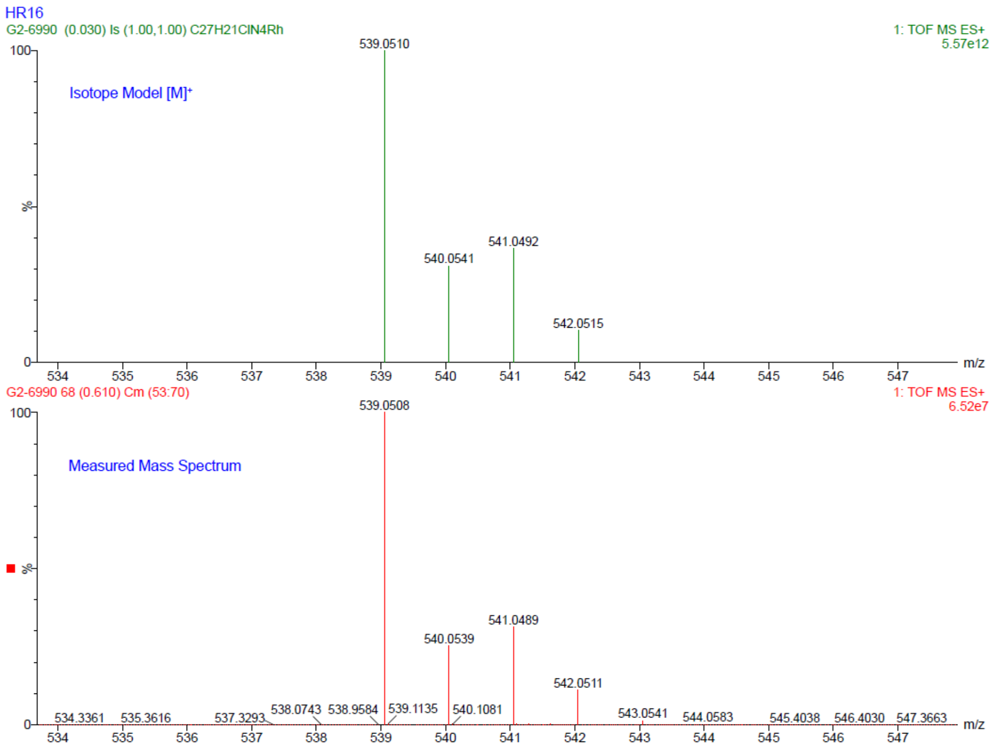

Figure S21. (Top) Theoretical isotope model for [**1**-PF_6]$^+$ ($C_{27}H_{21}ClN_4Rh$) and (bottom) the experimentally determined high-resolution ESI-TOF mass spectrum for complex **1**.

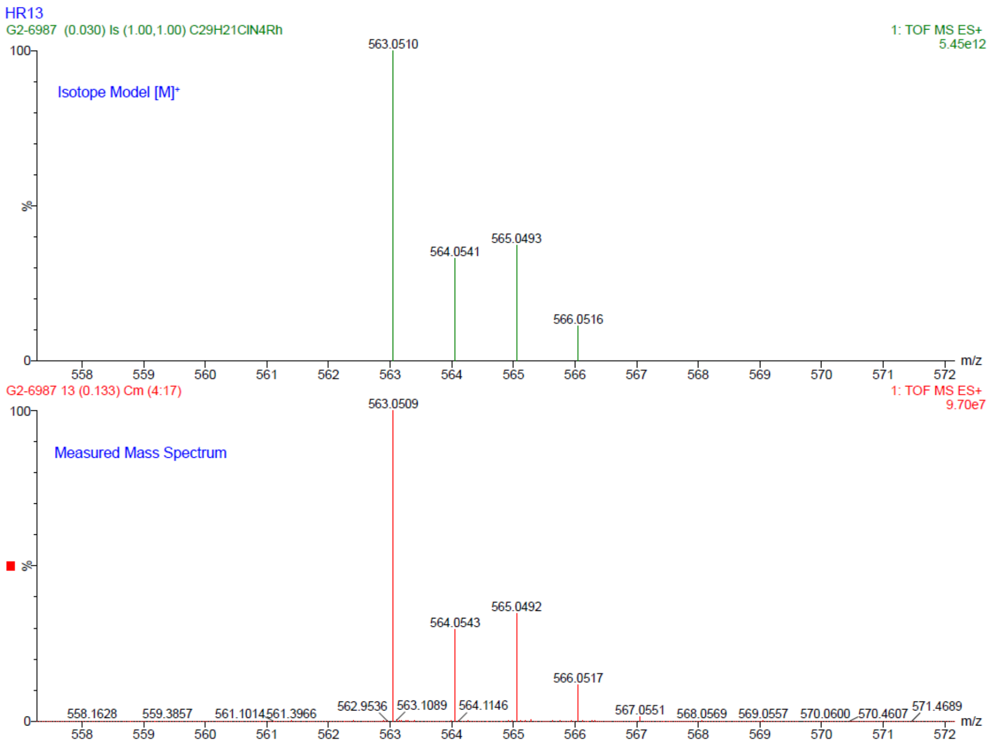

Figure S22. (Top) Theoretical isotope model for [**2**-PF_6]$^+$ ($C_{29}H_{21}ClN_4Rh$) and (bottom) the experimentally determined high-resolution ESI-TOF mass spectrum for complex **2**.

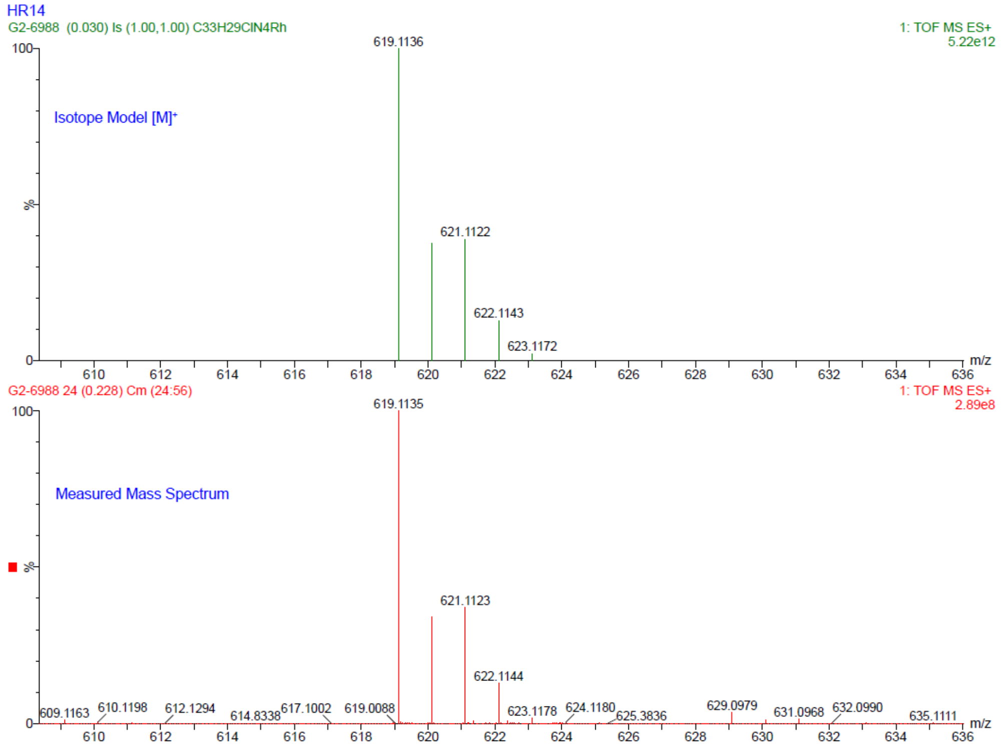

Figure S23. (Top) Theoretical isotope model for [**3**-PF_6]$^+$ ($C_{33}H_{29}ClN_4Rh$) and (bottom) the experimentally determined high-resolution ESI-TOF mass spectrum for complex **3**.

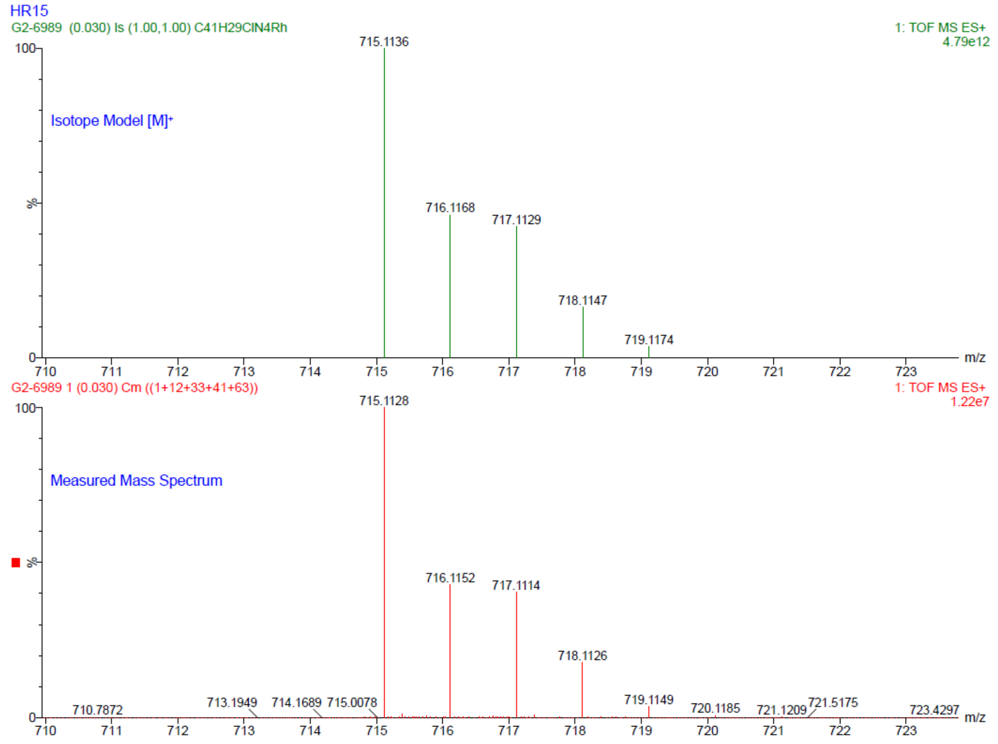

Figure S24. (Top) Theoretical isotope model for [**4**-PF_6]$^+$ ($C_{41}H_{29}ClN_4Rh$) and (bottom) the experimentally determined high-resolution ESI-TOF mass spectrum for complex **4**.

Table S1. Selected crystallographic data for complexes **1** and **2**.

**Metal complex**	**1**	**2**
CCDC No.	2518219	2518221
formula	$C_{27}H_{21}ClN_4RhPF_6 \cdot 2CH_2Cl_2$	$C_{29}H_{21}ClN_4RhPF_6$
F*w*	854.66	708.83
Crystal system	monoclinic	monoclinic
Space group	$P2_1/c$	$P2_1/n$
a, Å	11.2076(3)	15.2766(8)
b, Å	11.1452(3)	10.6004(6)
c, Å	26.1359(6)	16.7411(9)
α, deg.	90	90
β, deg.	90.4220(10)	96.320(2)
γ, deg.	90	90
V, $Å^3$	3264.57(14)	2694.5(3)
Z	4	4
$\rho_{calc} g/cm^3$	1.739	1.747
2θ / deg.	6.764 to 144.67	8.304 to 144.622
Reflections collected	25295	43583
Independent reflections	6349	5291 [
Goodness-of-fit on F^2	1.090	1.048
R_1, wR_2 [$I \geq 2\sigma(I)$]	.0417, 0.1074	0.0527, 0.1410
R_1, wR_2 [all data]	0.0424, 0.1078	0.0541, 0.1432
Largest diff. peak/hole / e $Å^{-3}$	1.08/-1.09	3.17/-1.50

Table S2. Selected crystallographic data for complexes **3** and **4**.

**Metal complex**	**3**	**4**
CCDC No.	2518220	2518222
formula	$C_{33}H_{29}ClN_4RhPF_6$	$C_{41}H_{29}ClN_4RhPF_6\cdot 3 C_2H_3N\cdot 2C_4H_{10}O$
F*w*	764.93	1132.41
Crystal system	monoclinic	orthorhombic
Space group	$P2_1/n$	$Pna2_1$
a, Å	12.6068(4)	10.9287(4)
b, Å	16.3083(5)	25.8934(8)
c, Å	15.2020(5)	18.0985(6)
α, deg.	90	90
β, deg.	95.136(2)	90
γ, deg.	90	90
V, $Å^3$	3112.91(17)	5121.5(3)
Z	4	4
$\rho_{calc} g/cm^3$	1.632	1.469
2θ / deg.	7.968 to 145.004	6.828 to 144.166
Reflections collected	35860	41700
Independent reflections	6148	9931
Goodness-of-fit on F^2	1.055	1.076
R_1, wR_2 [$I \geq 2\sigma(I)$]	0.0327, 0.0785	0.0397, 0.1037
R_1, wR_2 [all data]	0.0388, 0.0828	0.0457, 0.1075
Largest diff. peak/hole / e $Å^{-3}$	0.77/-0.63	1.36/-0.83

Table S3. Selected bond lengths (Å) and angles (°) for complex **1**.

Rh(1)-Cl(1)	2.3580(8)
Rh(1)-N(1)	2.108(3)
Rh(1)-N(2)	2.124(3)
Rh(1)-N(3)	2.043(3)
Rh(1)-N(4)	2.041(3)
Rh(1)-C(13)	2.029(3)
N(1)-Rh(1)-Cl(1)	91.58(8)
N(1)-Rh(1)-N(2)	78.02(12)
N(2)-Rh(1)-Cl(1)	86.62(8)
N(3)-Rh(1)-Cl(1)	177.61(9)
N(3)-Rh(1)-N(1)	90.46(11)
N(3)-Rh(1)-N(2)	92.60(11)
N(3)-Rh(1)-N(4)	87.17(11)
N(4)-Rh(1)-Cl(1)	90.75(8)
N(4)-Rh(1)-N(1)	177.07(11)
N(4)-Rh(1)-N(2)	100.35(12)
C(23)-Rh(1)-Cl(1)	90.75(9)
C(23)-Rh(1)-N(1)	96.72(12)
C(23)-Rh(1)-N(2)	174.03(12)
C(23)-Rh(1)-N(3)	90.24(12)
C(23)-Rh(1)-N(4)	85.02(13)

Table S4. Selected bond lengths (Å) and angles (°) for complex **2**.

Rh(1)-Cl(1)	2.3459(8)
Rh(1)-N(1)	2.082(3)
Rh(1)-N(2)	2.160(3)
Rh(1)-N(3)	2.044(3)
Rh(1)-N(4)	2.063(3)
Rh(1)-C(13)	2.010(3)
N(1)-Rh(1)-Cl(1)	87.59(8)
N(1)-Rh(1)-N(2)	78.92(12)
N(2)-Rh(1)-Cl(1)	85.52(7)
N(3)-Rh(1)-Cl(1)	91.32(8)
N(3)-Rh(1)-N(1)	176.88(11)
N(3)-Rh(1)-N(2)	98.08(11)
N(3)-Rh(1)-N(4)	87.90(11)
N(4)-Rh(1)-Cl(1)	179.04(7)
N(4)-Rh(1)-N(1)	93.16(11)
N(4)-Rh(1)-N(2)	94.05(10)
C(13)-Rh(1)-Cl(1)	91.88(9)
C(13)-Rh(1)-N(1)	96.97(12)
C(13)-Rh(1)-N(2)	175.21(12)
C(13)-Rh(1)-N(3)	85.98(12)
C(13)-Rh(1)-N(4)	88.62(11)

Table S5. Selected bond lengths (Å) and angles (°) for complex **3**.

Rh(1)-Cl(1)	2.3430(6)
Rh(1)-N(1)	2.115(2)
Rh(1)-N(2)	2.110(2)
Rh(1)-N(3)	2.041(2)
Rh(1)-N(4)	2.036(2)
Rh(1)-C(17)	2.025(2)
N(1)-Rh(1)-Cl(1)	87.22(7)
N(2)-Rh(1)-Cl(1)	88.09(6)
N(2)-Rh(1)-N(1)	78.59(9)
N(3)-Rh(1)-Cl(1)	178.52(7)
N(3)-Rh(1)-N(1)	94.15(9)
N(3)-Rh(1)-N(2)	91.63(9)
N(4)-Rh(1)-Cl(1)	91.26(7)
N(4)-Rh(1)-N(1)	97.19(9)
N(4)-Rh(1)-N(2)	175.76(9)
N(4)-Rh(1)-N(3)	89.13(10)
C(17)-Rh(1)-Cl(1)	90.72(7)
C(17)-Rh(1)-N(1)	176.36(9)
C(17)-Rh(1)-N(2)	98.35(9)
C(17)-Rh(1)-N(3)	87.88(10)
C(17)-Rh(1)-N(4)	85.85(10)

Table S6. Selected bond lengths (Å) and angles (°) for complex **4**.

Rh(1)-Cl(1)	2.3398(9)
Rh(1)-N(1)	2.084(3)
Rh(1)-N(2)	2.083(7)
Rh(1)-N(3)	2.160(7)
Rh(1)-N(4)	2.042(8)
Rh(1)-C(12)	1.967(9)
N(1)-Rh(1)-Cl(1)	178.7(3)
N(2)-Rh(1)-Cl(1)	90.84(16)
N(2)-Rh(1)-N(1)	88.0(3)
N(3)-Rh(1)-Cl(1)	86.18(15)
N(3)-Rh(1)-N(1)	93.4(3)
N(3)-Rh(1)-N(2)	96.8(3)
N(4)-Rh(1)-Cl(1)	89.72(16)
N(4)-Rh(1)-N(1)	91.4(3)
N(4)-Rh(1)-N(2)	175.9(3)
N(4)-Rh(1)-N(3)	79.20(13)
C(12)-Rh(1)-Cl(1)	91.6(2)
C(12)-Rh(1)-N(1)	88.9(3)
C(12)-Rh(1)-N(2)	85.90(16)
C(12)-Rh(1)-N(3)	176.5(3)
C(12)-Rh(1)-N(4)	98.1(4)

Table S7. Absorbance, emission (λ_{ex} = 340 nm), and quantum yield data for **1**–**4** (80 μM) in acetonitrile.

Compound	λ_{max} [nm] (ε [M^{-1} cm^{-1}])	λ_{em} [nm]	Φ [%]
**1**	301(12491)<br>311 (13527)<br>343 (1221)	378, 417, 515	0.35
**2**	271 (32054)<br>296 (12505)<br>313 (5921)<br>337 (2425)<br>354 (1863)	381, 416, 510	0.87
**3**	280 (30941)<br>299 (14026)<br>322 (5878)<br>338 (2411)<br>355 (858)	382, 409, 459	0.51
**4**	287 (49344)<br>321 (19129)<br>350 (6565)<br>370 (4427)	381, 438, 516	0.08

Table S8. Absorbance, emission (λ_{ex} = 340 nm), and quantum yield data for **1**–**4** (80 μM) in H_2O.

Compound	λ_{max} [nm] (ε [M^{-1} cm^{-1}])	λ_{em} [nm]	Φ [%]
**1**	301(12285)<br>312 (13055)<br>347 (722)	384, 420, 463	0.78
**2**	272 (26984)<br>297 (9680)<br>321 (2771)<br>334 (1760)<br>352 (1490)	384, 431, 467	0.67
**3**	280 (44712)<br>311 (12502)<br>323 (6668)<br>337 (3196)<br>354 (1284)	385, 410, 462	0.93
**4**	293 (45168)<br>334 (24193)<br>375 (11384)<br>430 (3871)	390, 419, 465	0.19

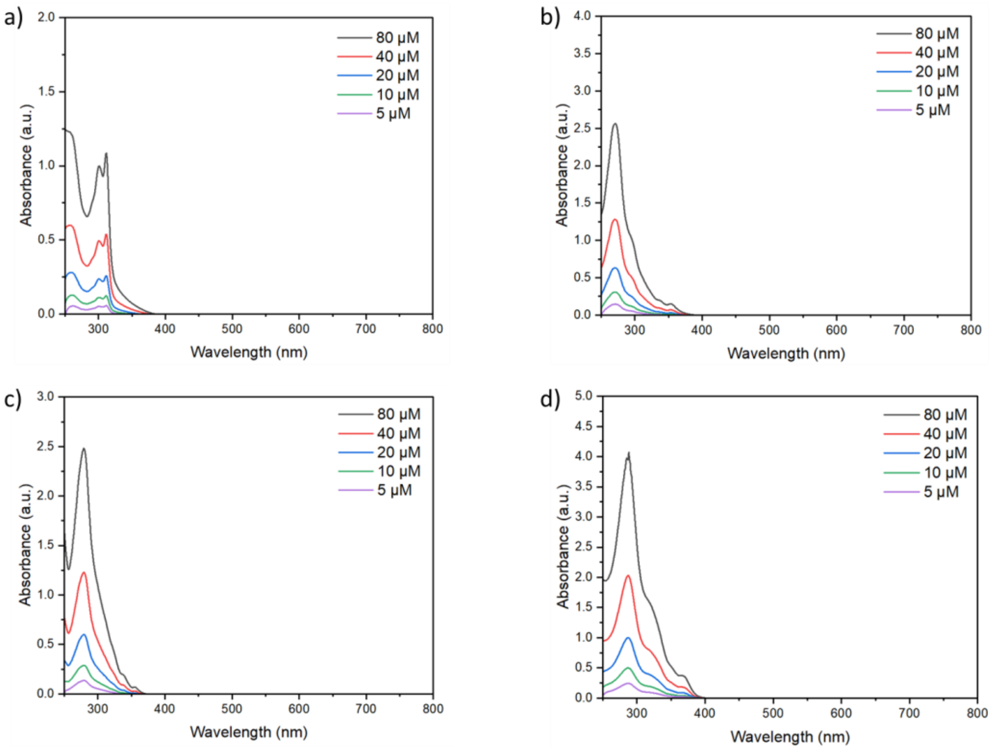

Figure S25. UV-vis spectra of (a) **1**, (b) **2**, (c) **3**, and (d) **4** (all 80 μM) in MeCN.

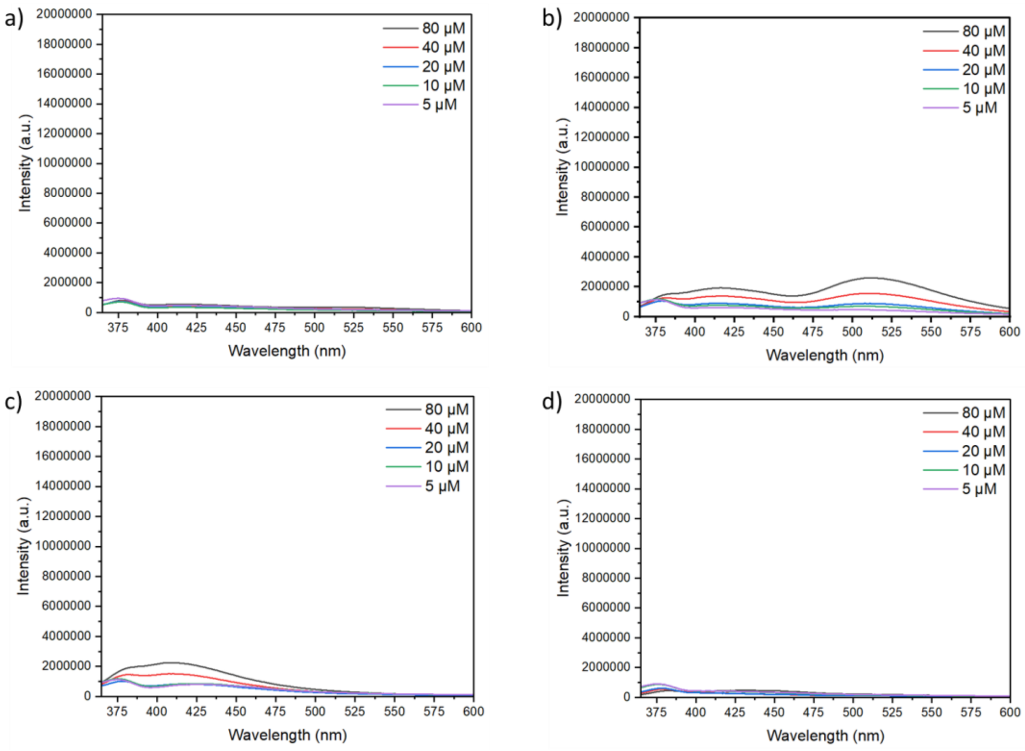

Figure S26. Fluorescence emission spectra of (a) **1**, (b) **2**, (c) **3**, and (d) **4** (all 80 μM) in MeCN (λ_{ex} = 340 nm).

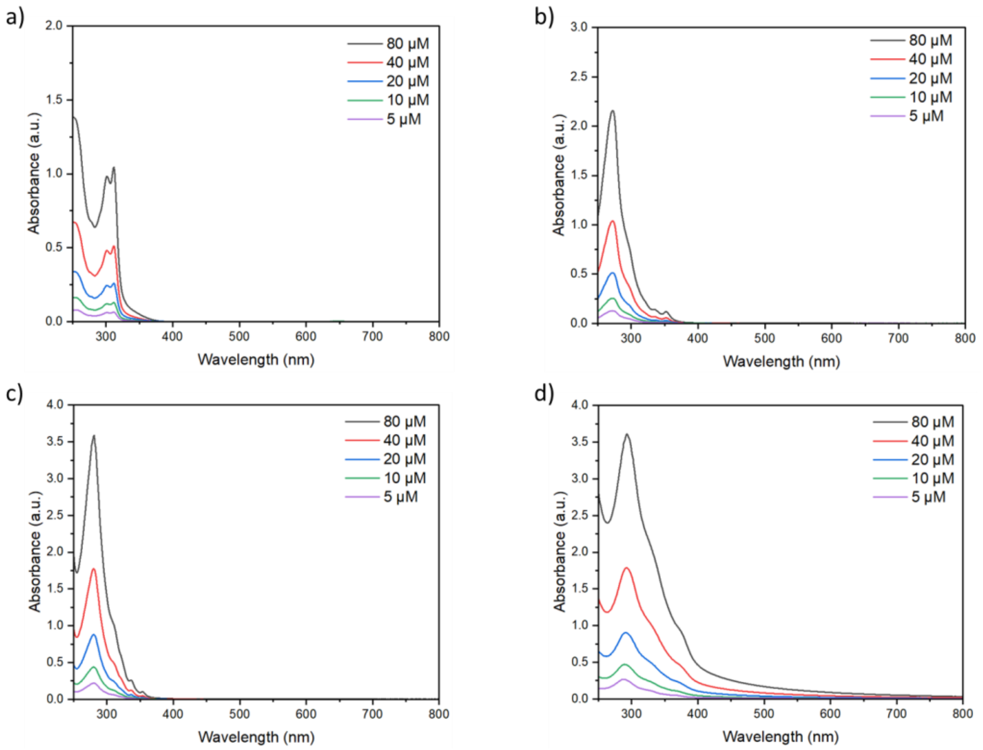

Figure S27. UV-vis spectra of (a) **1**, (b) **2**, (c) **3**, and (d) **4** (all 80 μM) in H_2O.

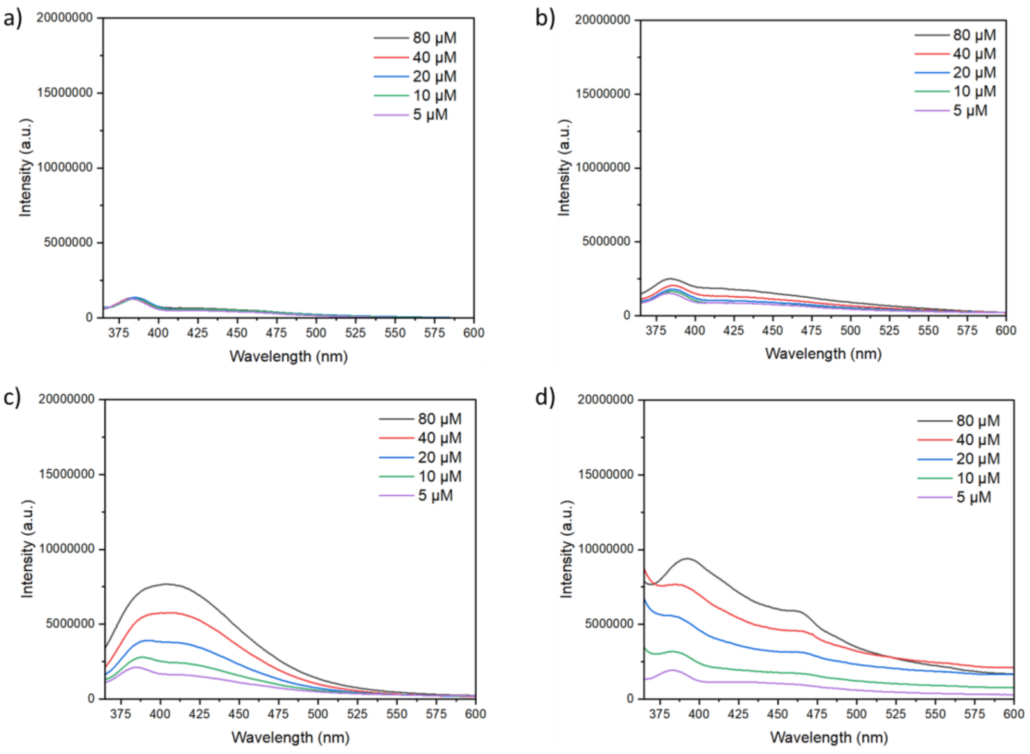

Figure S28. Fluorescence emission spectra of (a) **1**, (b) **2**, (c) **3**, and (d) **4** (all 80 μM) in H_2O (λ_{ex} = 340 nm).

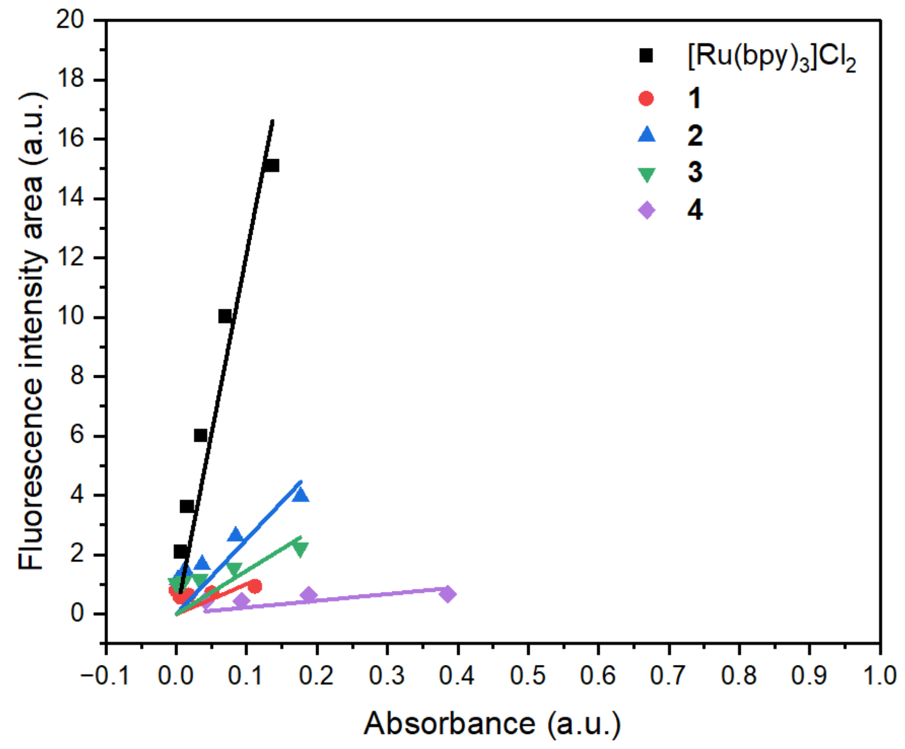

Figure S29. Fluorescence emission area versus absorption of **1-4** and [Ru(2,2'-bipyridine)$_3$]Cl_2 in MeCN (λ_{ex} = 340 nm). Used to calculate the quantum yield for **1-4**.

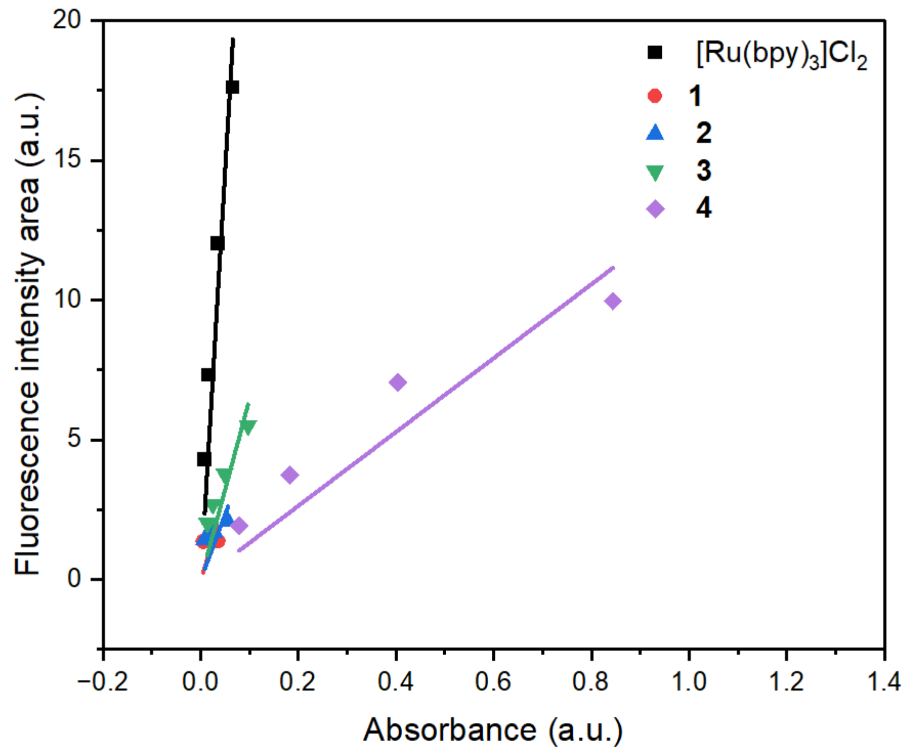

Figure S30. Fluorescence emission area versus absorption of **1-4** and [Ru(2,2'-bipyridine)$_3$]Cl_2 in H_2O (λ_{ex} = 340 nm). Used to calculate the quantum yield for **1-4**.

Table S9. Experimentally determined LogP values for **1-4**.

Rh(III) complex	LogP
**1**	-0.88 ± 0.01
**2**	0.07 ± 0.03
**3**	0.18 ± 0.02
**4**	1.50 ± 0.08

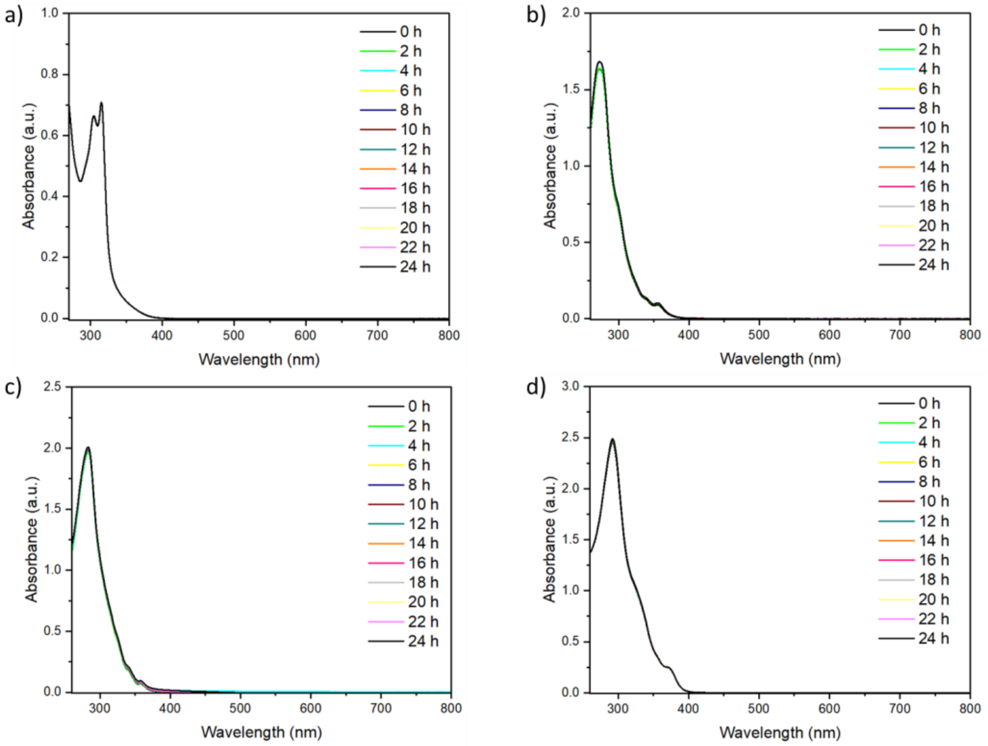

Figure S31. UV-vis spectra of (a) **1**, (b) **2**, (c) **3** or (d) **4** (all 50 µM) in DMSO over the course of 24 h at 37 °C.

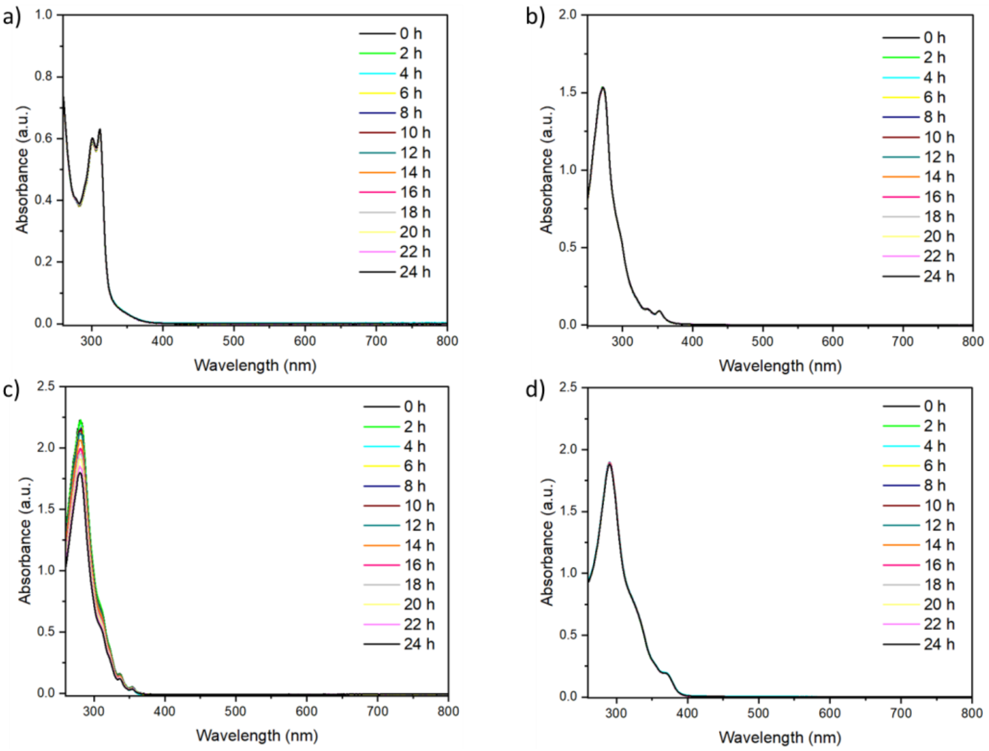

Figure S32. UV-vis spectra of (a) **1**, (b) **2**, (c) **3** or (d) **4** (all 50 µM) in DMEM:DMSO (200:1 or 1:1) over the course of 24 h at 37 °C.

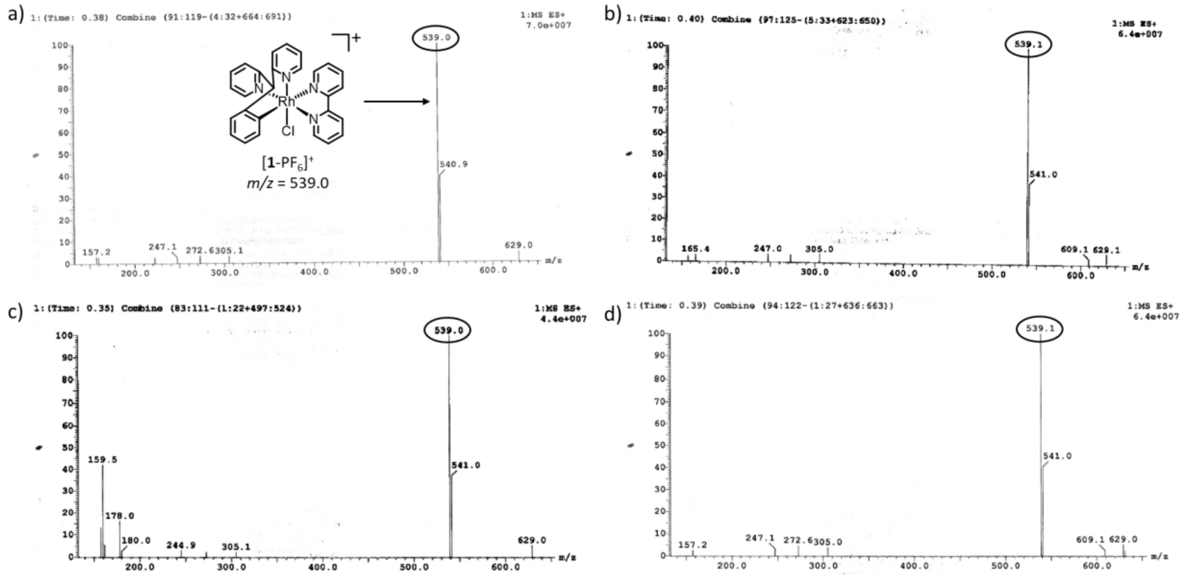

Figure S33. ESI mass spectra of **1** (40 µM) in H_2O:DMSO (125:1) at 37 °C after (a) 0 h, (b) 24 h, (c) 48 h or (d) 72 h incubation.

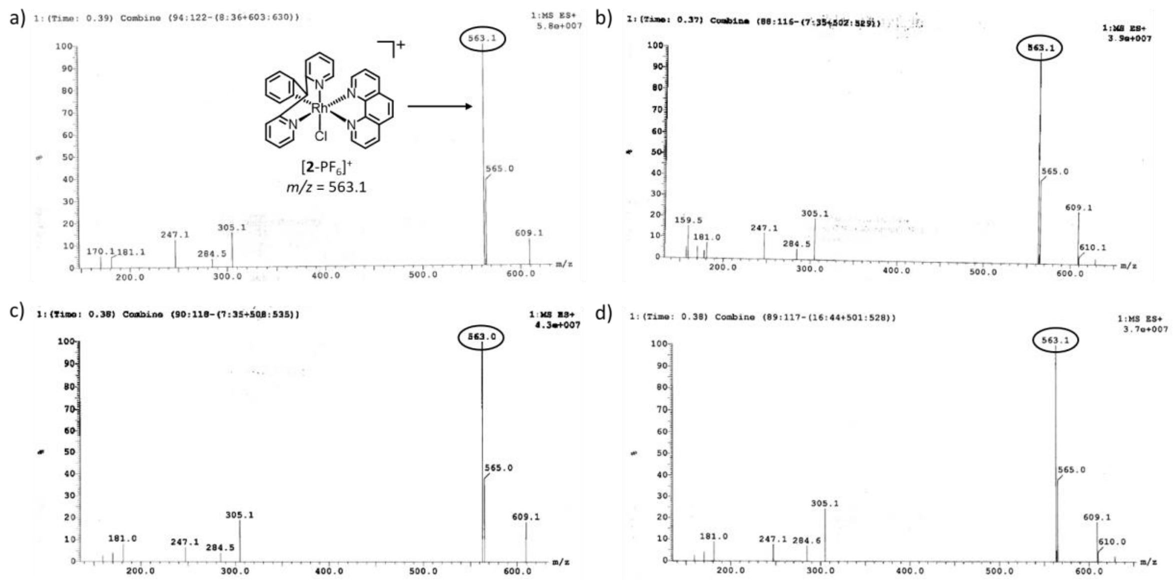

Figure S34. ESI mass spectra of **2** (40 μM) in H_2O:DMSO (125:1) at 37 °C after (a) 0 h, (b) 24 h, (c) 48 h or (d) 72 h incubation.

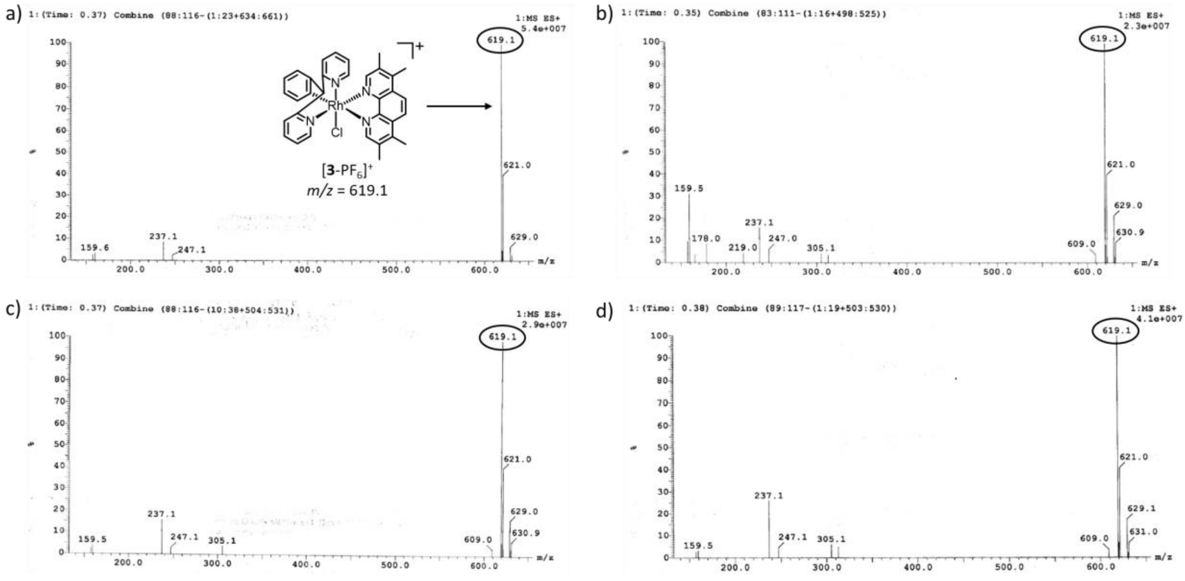

Figure S35. ESI mass spectra of **3** (40 μM) in H_2O:DMSO (125:1) at 37 °C after (a) 0 h, (b) 24 h, (c) 48 h or (d) 72 h incubation.

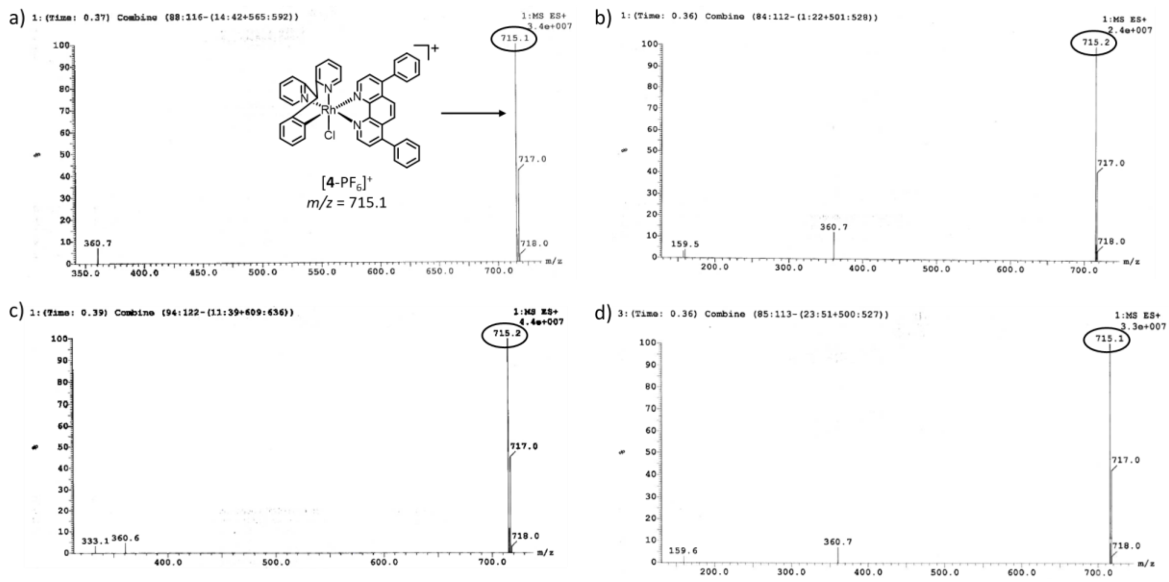

Figure S36. ESI mass spectra of **4** (40 µM) in H_2O:DMSO (125:1) at 37 °C after (a) 0 h, (b) 24 h, (c) 48 h or (d) 72 h incubation.

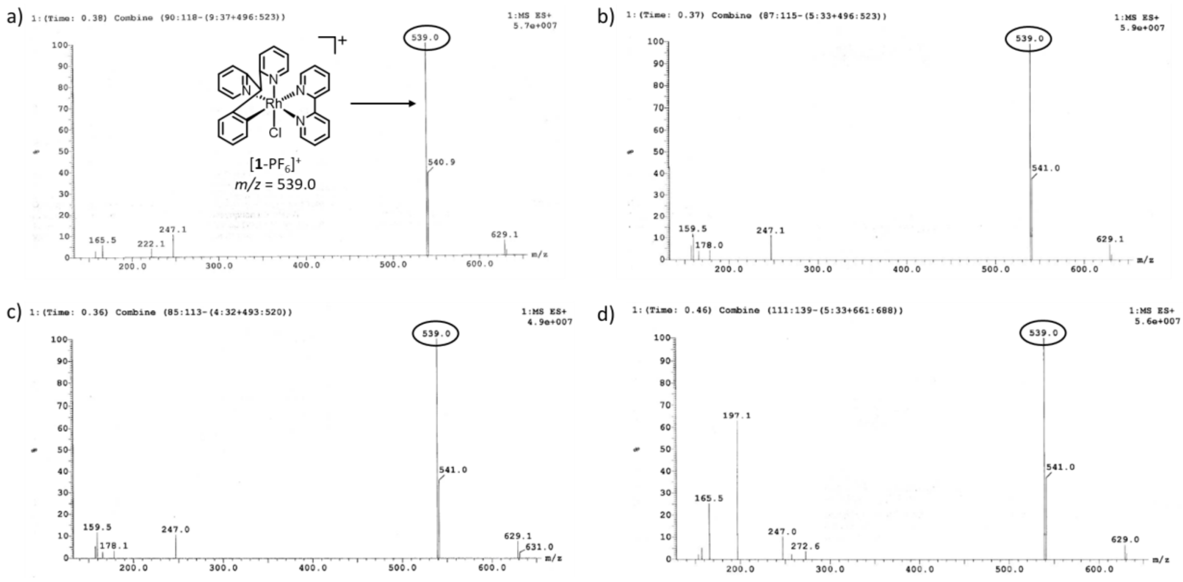

Figure S37. ESI mass spectra of **1** (40 µM) in H_2O:DMSO (125:1) in the presence of ascorbic acid (400 µM) at 37 °C after (a) 0 h, (b) 24 h, (c) 48 h or (d) 72 h incubation.

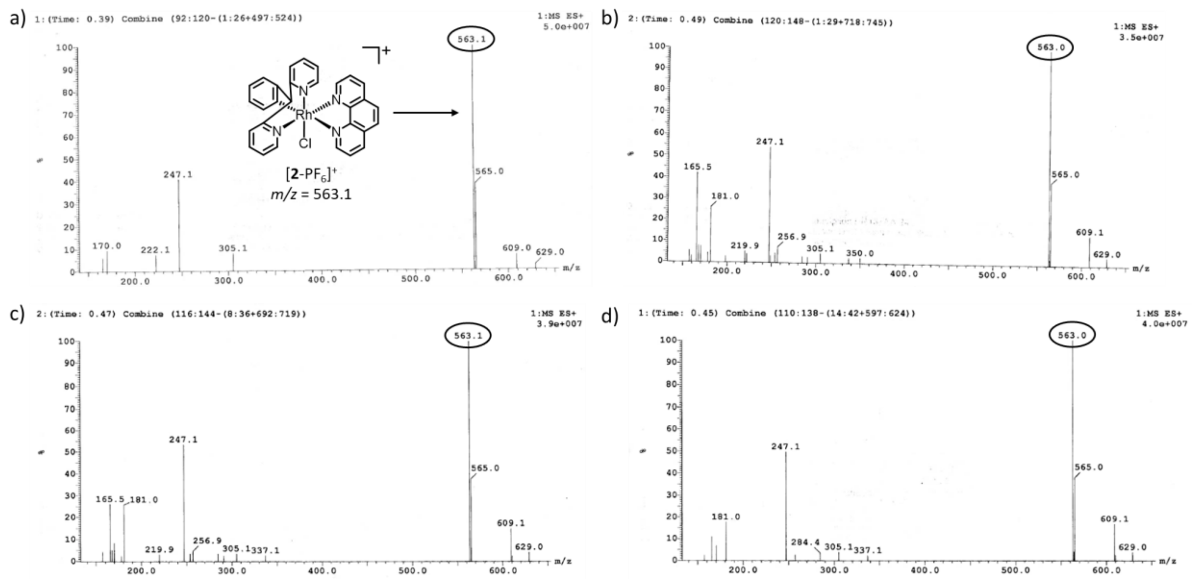

Figure S38. ESI mass spectra of **2** (40 μM) in H_2O:DMSO (125:1) in the presence of ascorbic acid (400 μM) at 37 °C after (a) 0 h, (b) 24 h, (c) 48 h or (d) 72 h incubation.

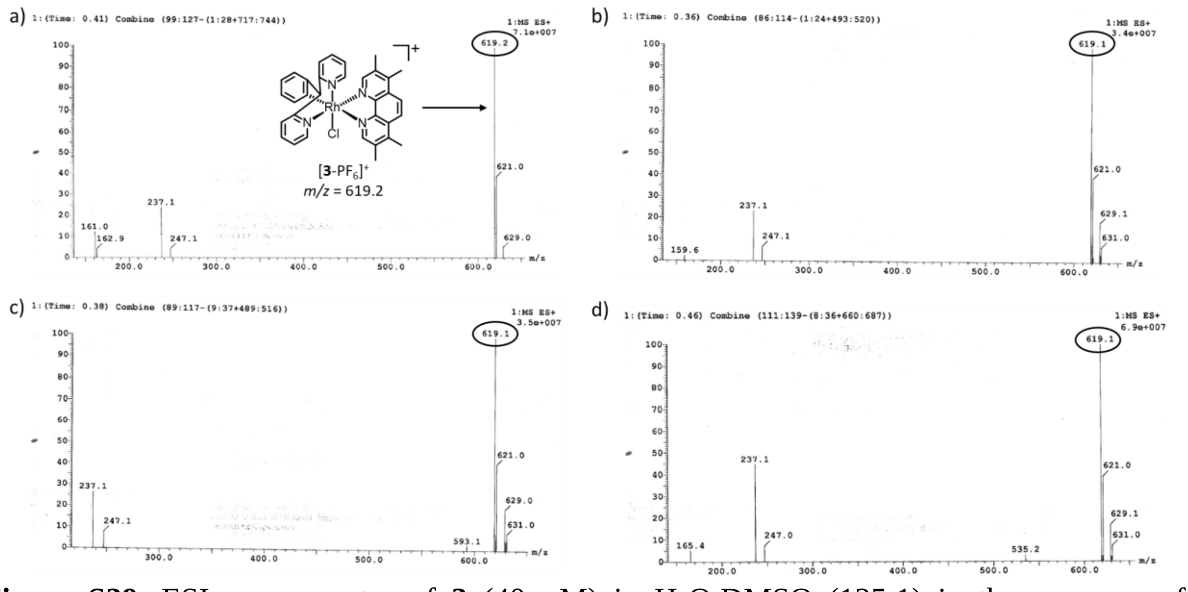

Figure S39. ESI mass spectra of **3** (40 μM) in H_2O:DMSO (125:1) in the presence of ascorbic acid (400 μM) at 37 °C after (a) 0 h, (b) 24 h, (c) 48 h or (d) 72 h incubation.

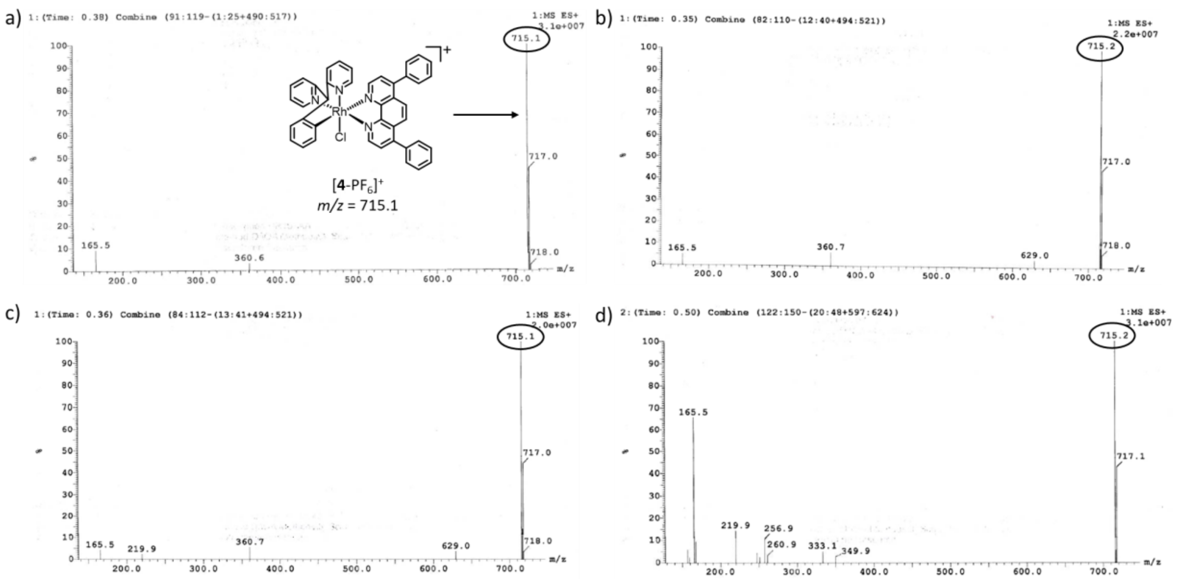

Figure S40. ESI mass spectra of **4** (40 µM) in H_2O:DMSO (125:1) in the presence of ascorbic acid (400 µM) at 37 °C after (a) 0 h, (b) 24 h, (c) 48 h or (d) 72 h incubation.

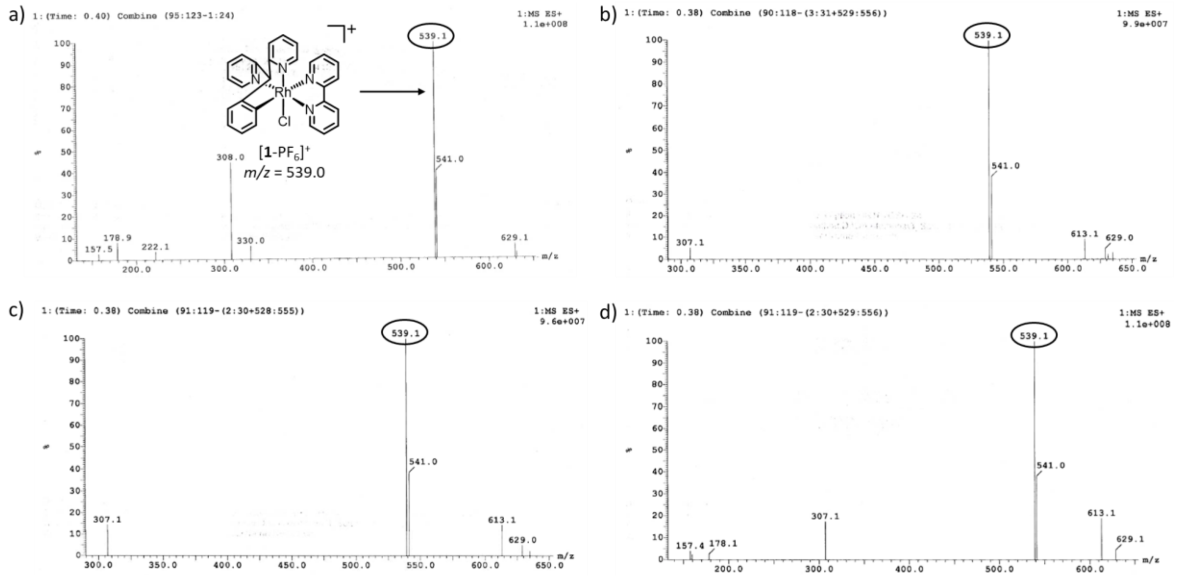

Figure S41. ESI mass spectra of **1** (40 µM) in H_2O:DMSO (125:1) in the presence of glutathione (400 µM) at 37 °C after (a) 0 h, (b) 24 h, (c) 48 h or (d) 72 h incubation.

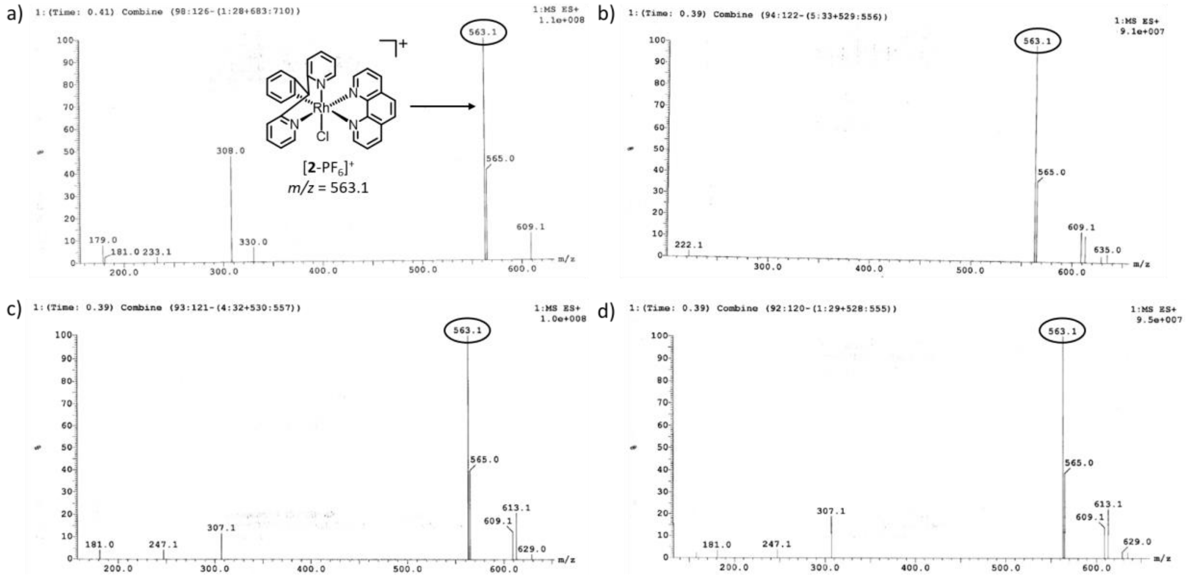

Figure S42. ESI mass spectra of **2** (40 μM) in H_2O:DMSO (125:1) in the presence of glutathione (400 μM) at 37 °C after (a) 0 h, (b) 24 h, (c) 48 h or (d) 72 h incubation.

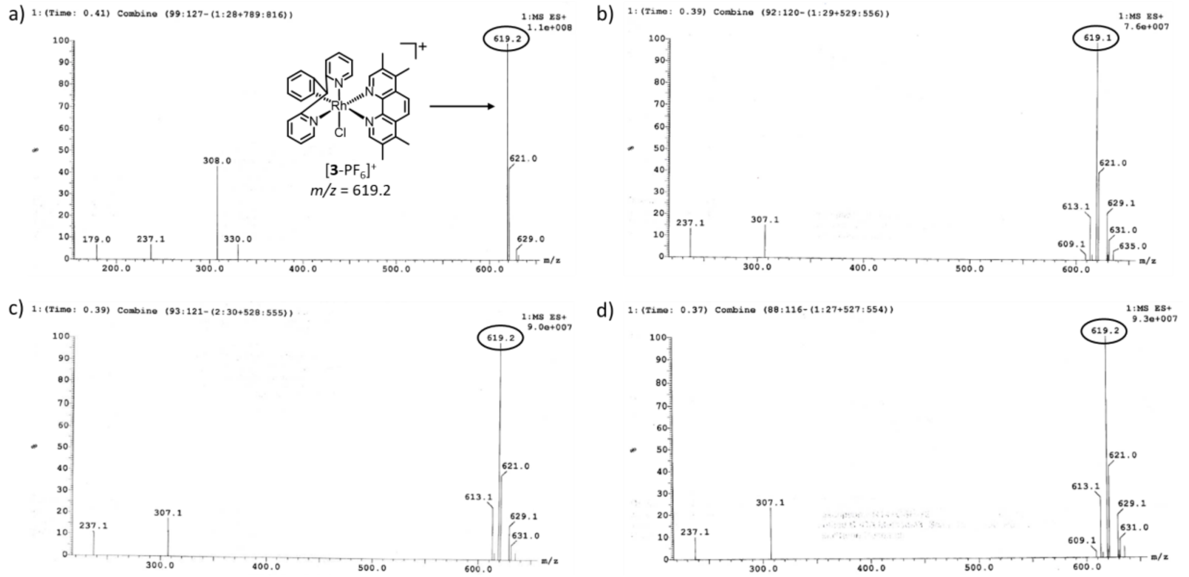

Figure S43. ESI mass spectra of **3** (40 μM) in H_2O:DMSO (125:1) in the presence of glutathione (400 μM) at 37 °C after (a) 0 h, (b) 24 h, (c) 48 h or (d) 72 h incubation.

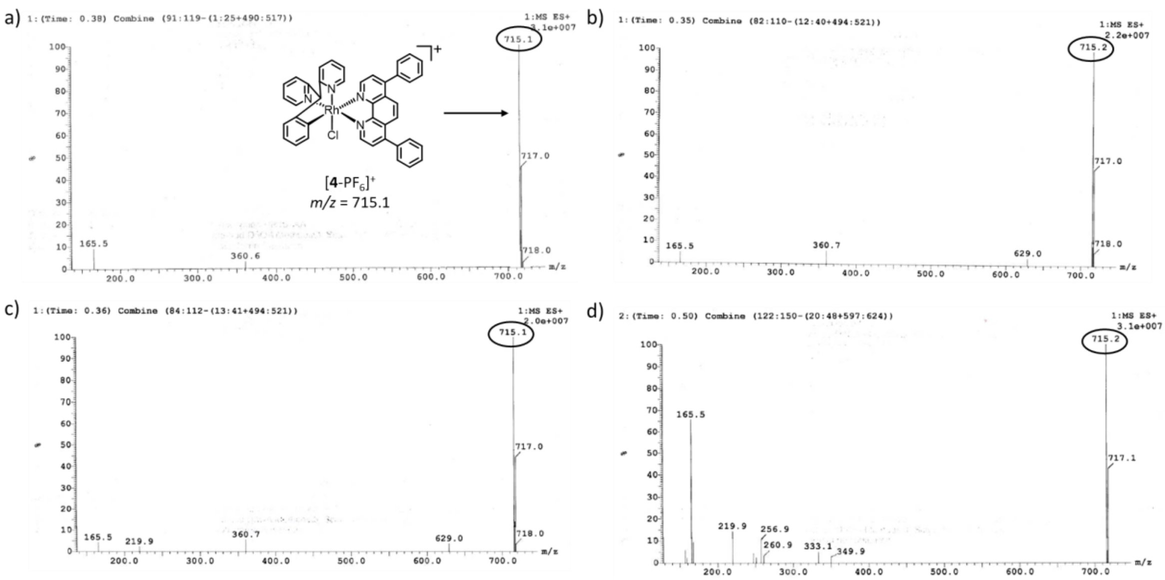

Figure S44. ESI mass spectra of **4** (40 µM) in H_2O:DMSO (125:1) in the presence of glutathione (400 µM) at 37 °C after (a) 0 h, (b) 24 h, (c) 48 h or (d) 72 h incubation.

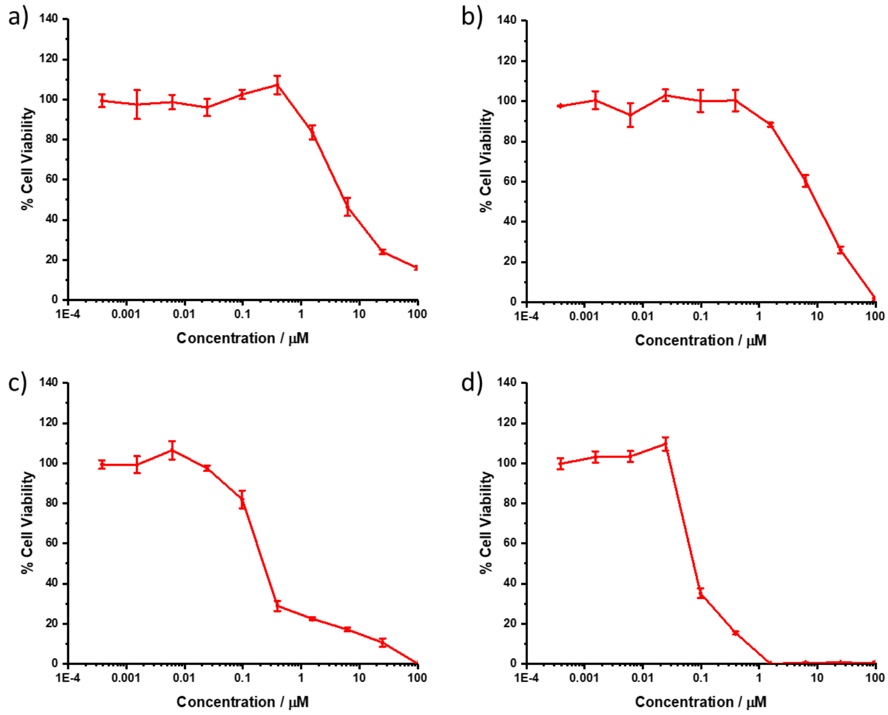

Figure S45. Representative dose-response curves for the treatment of HMLER cells with (a) **1**, (b) **2**, (c) **3** or (d) **4** after 72 h incubation.

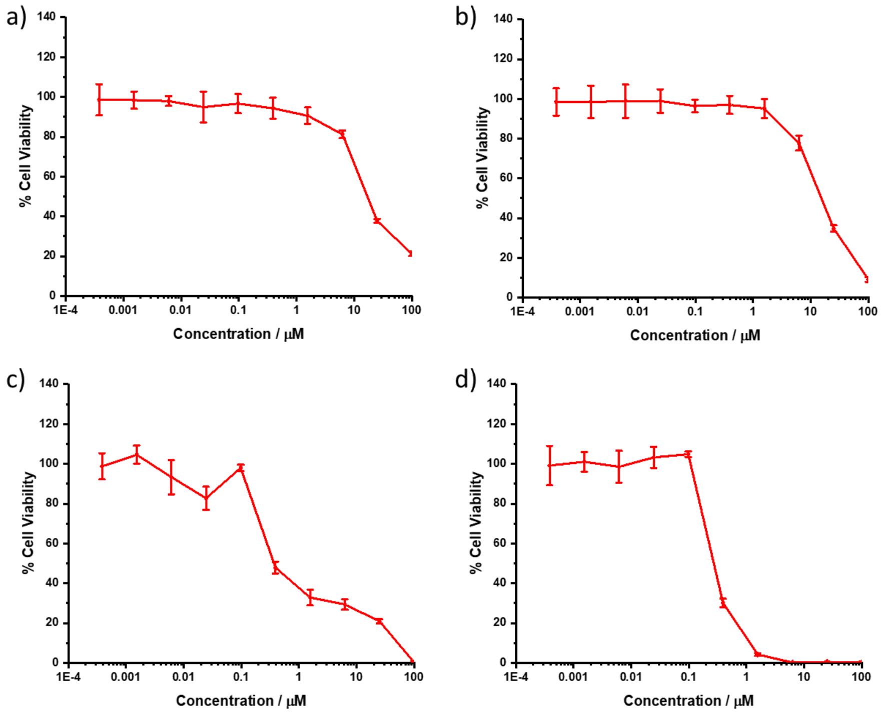

Figure S46. Representative dose-response curves for the treatment of HMLER-shEcad cells with (a) **1**, (b) **2**, (c) **3** or (d) **4** after 72 h incubation.

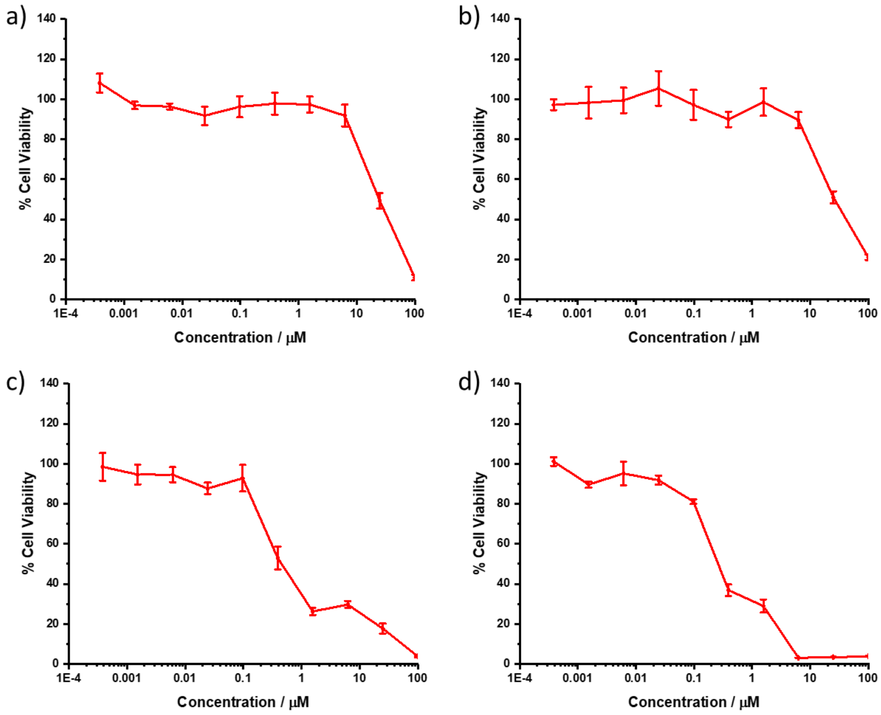

Figure S47. Representative dose-response curves for the treatment of U2OS cells with (a) **1**, (b) **2**, (c) **3** or (d) **4** after 72 h incubation.

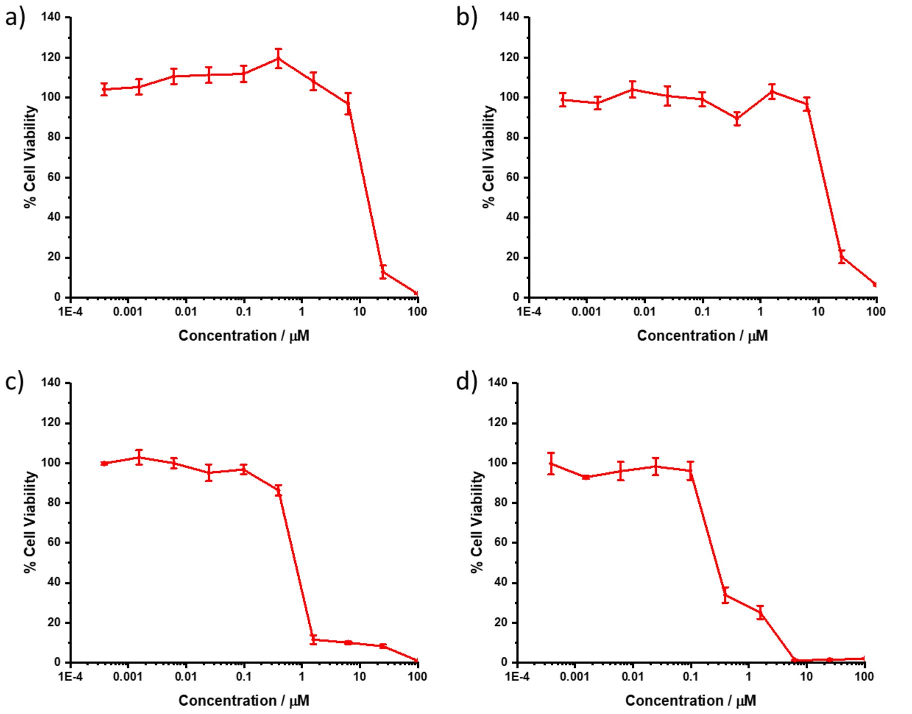

Figure S48. Representative dose-response curves for the treatment of USOS-MTX cells with (a) **1**, (b) **2**, (c) **3** or (d) **4** after 72 h incubation.

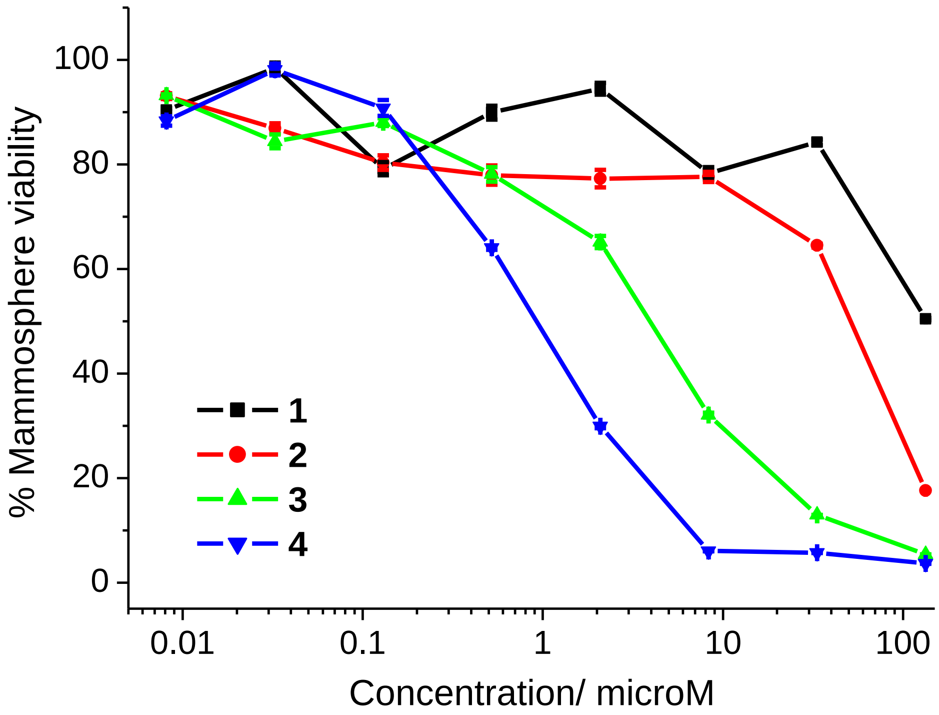

Figure S49. Representative dose-response curves for the treatment of HMLER-shEcad mammospheres with **1-4** after 5 days incubation.

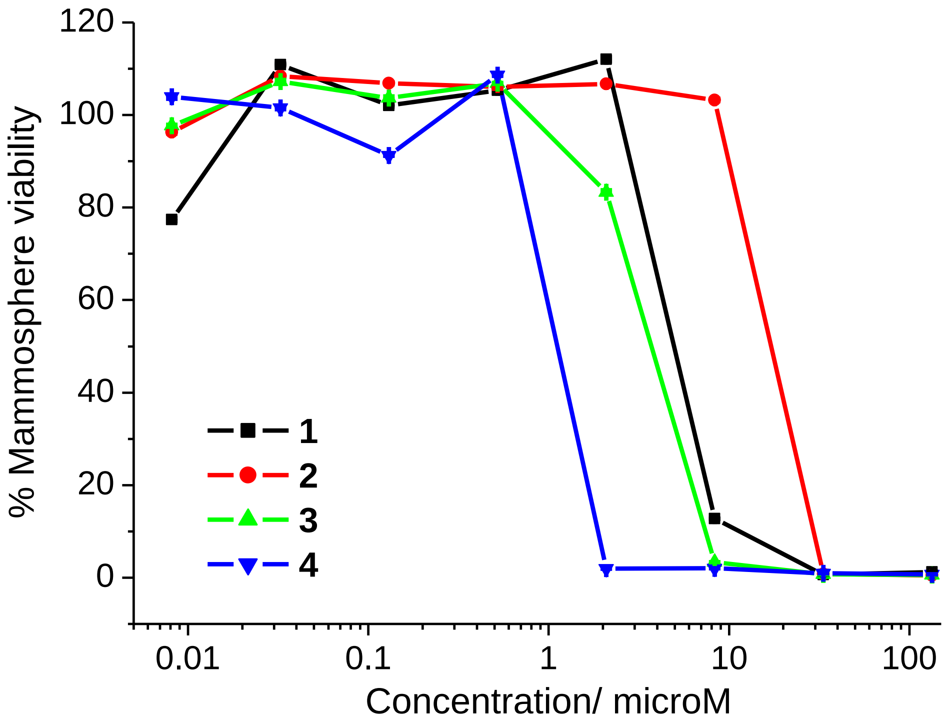

Figure S50. Representative dose-response curves for the treatment of U2OS-MTX sarcospheres with **1-4** after 10 days incubation.

References

(1) (a) Hierlinger, C.; Cordes, D. B.; Slawin, A. M. Z.; Jacquemin, D.; Guerchais, V.; Zysman-Colman, E. Phosphorescent cationic iridium(III) complexes bearing a nonconjugated six-membered chelating ancillary ligand: a strategy for tuning the emission towards the blue. *Dalton Trans.* **2018**, *47* (31), 10569-10577. (b) Ji, X.; Huang, T.; Wu, W.; Liang, F.; Cao, S. LDA-Mediated Synthesis of Triarylmethanes by Arylation of Diarylmethanes with Fluoroarenes at Room Temperature. *Org. Lett.* **2015**, *17* (20), 5096-5099. (c) Jones, M. R.; Fast, C. D.; Schley, N. D. Iridium-Catalyzed sp^3 C–H Borylation in Hydrocarbon Solvent Enabled by 2,2′-Dipyridylarylmethane Ligands. *J. Am. Chem. Soc.* **2020**, *142* (14), 6488-6492.

(2) Sheldrick, G. M., *Program for Area Detector Absorption Correction, Institute for Inorganic Chemistry*; University of Göttingen: Göttingen, Germany, **1996**.

(3) Sheldrick, G. A short history of SHELX. *Acta Cryst.* **2008**, *A64* (1), 112-122.

(4) Sheldrick, G. M. Crystal structure refinement with SHELXL. *Acta Crystallogr. Sect. C* **2015**, *71* (Pt 1), 3-8.

(5) Dolomanov, O. V.; Bourhis, L. J.; Gildea, R. J.; Howard, J. A. K.; Puschmann, H.: a complete structure solution, refinement and analysis program. *J. Appl. Crystallogr.* **2009**, *42*, 339-341.

(6) Robin, P.; Singh, K.; Suntharalingam, K. Gallium(III)-polypyridyl complexes as anti-osteosarcoma stem cell agents. *Chem. Commun.* **2020**, *56* (10), 1509-1512.
